# Bioinspired Lubricity from Surface Gel Layers

**DOI:** 10.1021/acs.langmuir.3c03686

**Published:** 2024-04-29

**Authors:** Ahmed Al Kindi, Nemea S. Courelli, Kevin Ogbonna, Juan Manuel Urueña, Allison L. Chau, Angela A. Pitenis

**Affiliations:** † Department of Mechanical Engineering, University of California, Santa Barbara, California 93106, United States; ‡ Department of Chemical Engineering, University of California, Santa Barbara, California 93106, United States; § College of Creative Studies, Biological Sciences, University of California, Santa Barbara, California 93106, United States; ∥ NSF BioPACIFIC Materials Innovation Platform, University of California, Santa Barbara, California 93106, United States; ⊥ Materials Department, University of California, Santa Barbara, California 93106, United States

## Abstract

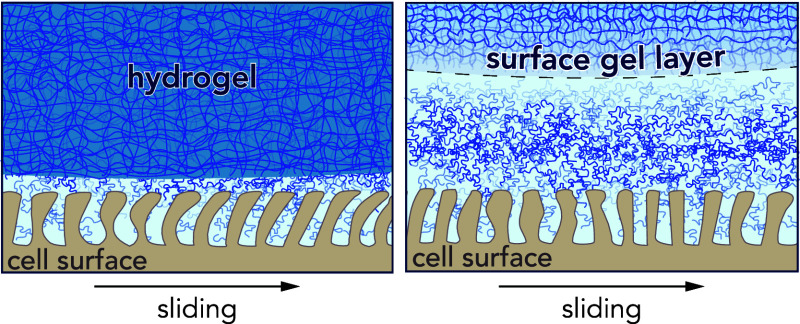

Surface gel layers on commercially available contact lenses have been shown to reduce frictional shear stresses and mitigate damage during sliding contact with fragile epithelial cell layers in vitro. Spencer and co-workers recently demonstrated that surface gel layers could arise from oxygen-inhibited free-radical polymerization. In this study, polyacrylamide hydrogel shell probes (7.5 wt % acrylamide, 0.3 wt % *N,N'*-methylenebisacrylamide) were polymerized in three hemispherical molds listed in order of decreasing surface energy and increasing oxygen permeability: borosilicate glass, polyether ether ketone (PEEK), and polytetrafluoroethylene (PTFE). Hydrogel probes polymerized in PEEK and PTFE molds exhibited 100× lower elastic moduli at the surface ( = 80 ± 31 and  = 106 ± 26 Pa, respectively) than those polymerized in glass molds ( = 31,560 ± 1,570 Pa), in agreement with previous investigations by Spencer and co-workers. Biotribological experiments revealed that hydrogel probes with surface gel layers reduced frictional shear stresses against cells (*τ*_PEEK_ = 35 ± 15 and *τ*_PTFE_ = 22 ± 16 Pa) more than those without (*τ*_glass_ = 68 ± 15 Pa) and offered greater protection against cell damage when sliding against human telomerase-immortalized corneal epithelial (hTCEpi) cell monolayers. Our work demonstrates that the “mold effect” resulting in oxygen-inhibition polymerization creates hydrogels with surface gel layers that reduce shear stresses in sliding contact with cell monolayers, similar to the protection offered by gradient mucin gel networks across epithelial cell layers.

## Introduction

The protective aspis shielding the body’s first line of defense are fragile biopolymer gels.^1^ These aqueous networks, composed of heavily glycosylated polypeptides (mucins), are continuously secreted by moist epithelial cells lining all external interfaces in the body (e.g., the eye, respiratory tract, gastrointestinal tract, reproductive tract, pleural cavity, organ sheaths, etc.).^2−5^ Mucin gels serve multiple functions, including physical barriers to pathogens and debris,^6−8^ moisture-retaining layers,^9−11^ and mechanical fuses capable of supporting loads at rest, yet shear-thinning and rapidly rehealing in response to low shear stress.^12−14^ In the eye, mucin gels are secreted by opposing corneal and conjunctival epithelium that together create a gel-spanning network across the ocular tear film with shallow gradients of mucin emanating from each tissue layer.^15−18^ Biomedical devices and implants placed directly against delicate mucin gels to improve form or function can disrupt natural biological lubrication mechanisms and lead to discomfort (e.g., contact lens-induced dry eye^19−22^). Over the past decade, prevalent design strategies to reduce device-induced discomfort have involved weak interfacial layers inspired by mucin gels. Such approaches range from inexpensive and imprecise application of aqueous gels to device surfaces prior to invasive procedures (e.g., specula,^23,24^ ventilators,^25−27^ laparoscopes^28,29^) to costly and exacting chemical synthesis of interpenetrating networks for the robust integration and attachment of surface gel layers to devices (e.g., gradient gel contact lenses^30−33^). Reducing the complexity and expenses associated with lowering friction across device-tissue interfaces could potentially mitigate device-associated infections and improve patient outcomes.^34−36^

Recent investigations by Spencer and co-workers^37−39^ have opened new avenues for the creation of fragile gel networks extending from bulk hydrogels owing to their processing conditions that hold promise for broader application in biomedical devices. Inspired by the pioneering work of Gong and others that established the “mold effect” of hydrogels polymerized near hydrophobic materials,^40,41^ Spencer, Simic, Gombert, and coauthors elegantly demonstrated that hydrogels polymerized near low surface energy materials (e.g., PTFE and PEEK)^39^ are also oxygen-rich interfaces^37^ and thus exhibit gradients in polymer density and higher water content toward the surface.^42^ Furthermore, Spencer and co-workers^38^ demonstrated that these surface gel layers reduce both elastic modulus and the coefficient of friction over a wide range of sliding conditions (e.g., contact pressure, sliding velocity) and configurations (e.g., migrating/stationary/Gemini contact^43^). Contact mechanics approaches developed by Dunn and co-workers enabled estimations of the thickness of oxygen-inhibited surface gel layers ranging from single to tens of micrometers.^44−47^ Reaction-kinetics models of peroxidation gradients at air–liquid interfaces agreed with these findings and further proposed surface gel layer thickness and their friction-reducing capabilities could be controlled with oxygen concentration and polymerization time.^48^

In vitro investigations modeling device-tissue sliding interfaces demonstrated that surface gel layers on commercially available contact lenses reduce frictional shear stress and damage to human telomerase-immortalized corneal epithelial (hTCEpi) cell layers.^14^ Sawyer and co-workers used microrheology to estimate the water content of contact lens surface gel layers to be above 90% and their elastic modulus to be below 1 kPa.^49^ Earlier work demonstrated that increasing the water content of polyacrylamide hydrogel probes reduced frictional shear stress and cell death in hTCEpi cell monolayers^50,51^ and designing spherical shell hydrogel probes with different geometries could control the frictional shear stress through contact pressure.^52,53^ However, open questions remain regarding the extent to which surface gel layers could reduce friction against cell monolayers from the “mold effect” alone. In this work, spherical shell hydrogel probes were polymerized in the same ambient conditions from the same hydrogel precursor solution and in the same mold geometries, but polymerized against three different mold materials with decreasing surface energy, γ, and increasing oxygen permeability, *k*: borosilicate glass (γ = 64 mN m^–1^*; k* ≈ 0*)*,^39,54^ polyetheretherketone (PEEK) (γ= 33.5 mN m^–1^; *k* = 0.13 barrer*)*,^54,55^ and polytetrafluoroethylene (PTFE) (γ = 19 mN m^–1^; *k* = 4.2 barrer*)*.^54,56^ The values for permeability are reported in barrer units, where 1 barrer = 3.35 × 10^–16^ mol m m^–2^ s^–1^ Pa^–1^. Hydrogel probes were used in biotribological studies against hTCEpi cell monolayers and evaluated for their ability to reduce frictional shear stresses and gently interact with—as opposed to disrupting and removing—the natural mucin gels at the sliding interface. The results from this investigation agree with prior work and suggest that hydrogel surface gel layers from oxygen gradients are an effective approach for bioinspired lubrication against living cells and tissues.

## Materials and Methods

### Hydrogel Sample Preparation

Hydrogels were prepared in ambient conditions by combining 7.5 wt % acrylamide (AAm), 0.3 wt % *N,N′*-methylenebis(acrylamide) (MBAm), 0.15 wt % ammonium persulfate (APS), and 0.15 wt % tetramethylethylenediamine (TEMED) in ultrapure water (18.2 MΩ-cm resistivity). Each spherical shell hydrogel probe was polymerized from 80 μL of this solution following the methods in Marshall et al.^53^ The shell probe was created from a backing composed of polyoxymethylene (POM). The hydrogels polymerized for 3–5 min before removal from the mold. Hemispherical molds composed of borosilicate glass (Wilmad-LabGlass part number 669995800) as well as custom-designed molds composed of either polyetheretherketone (PEEK) or polytetrafluoroethylene (PTFE) manufactured by Ocular Technology Inc. in Goleta, California were used. The manufacturer-specified surface roughness of the PEEK and PTFE molds was better than Ra < 1 μm. The radius of curvature of all hydrogel probes was *R*_curvature_ = 2 mm, and the thickness of the apical surface of the probes was *t*_shell_ ≈ 250 μm (Supporting Information Section 1). The radius of curvature measurements were acquired by imaging a selection of hydrogel probes. Sectioning probes in half with sharp blades enabled direct observations of the apical shell thickness from optical microscopy. Hydrogel probes were equilibrated in ultrapure water for at least 24 h and then equilibrated in phosphate-buffered saline (PBS, 1× conc.) for a minimum of 24 h prior to testing.

### Cell Culture

Human telomerase-immortalized corneal epithelial cells were gifted by Prof. James Jester from UC Irvine^57^ and used between passage numbers 55 and 74. These cells are known to secrete multiple types of mucins, including MUC1, MUC4, and MUC16.^58^ MUC1 Recombinant Anti-MUC1 antibody [EP1024Y] (Abcam ab45167) and recombinant Anti-MUC16 antibody [EPSISR23]—BSA and Azide free (Abcam ab271903) were used to identify MUC1 and MUC16, respectively (Supporting Information Section 2). Cells were cultured in T75 polystyrene flasks (ThermoFisher Cat. no. 156800) in KGM Gold growth media and supplemented with BulletKit (Lonza Basel Cat. no. NC0230946). Prior to tribological testing, cells were plated on fibronectin-coated glass-bottom culture dishes (Fisher Scientific Nunc Cat. no. 150680) and grown to approximately 80% confluence (Supporting Information Section 3). Cells were stained using CellTracker Green CMFDA Dye (Cat. no . C7025; conc. 5 μM), CellTracker Orange CMTMR Dye (Cat. no. C2927; conc. 5 μM), and CellTracker Deep Red Dye (Cat. no. C34565; conc. 1 μM) to monitor cell viability (Supporting Information Section 4). Cells were also stained with wheat germ agglutinin (WGA) (conc. 0.085 μM, Alexa Fluor 488), and propidium iodide (conc. 2.39 nM), to measure the relative abundance of mucin gels and monitor cell death, respectively. Corneal mucin is routinely quantified in clinical settings using WGA.^9,59^ Throughout experiments, hTCEpi cells were maintained under homeostatic conditions (37 °C, > 95% relative humidity, 5% CO_2_) using a custom-built incubator mounted to the microscope platform.

### Confocal Microtribometer

**Figure 1:**
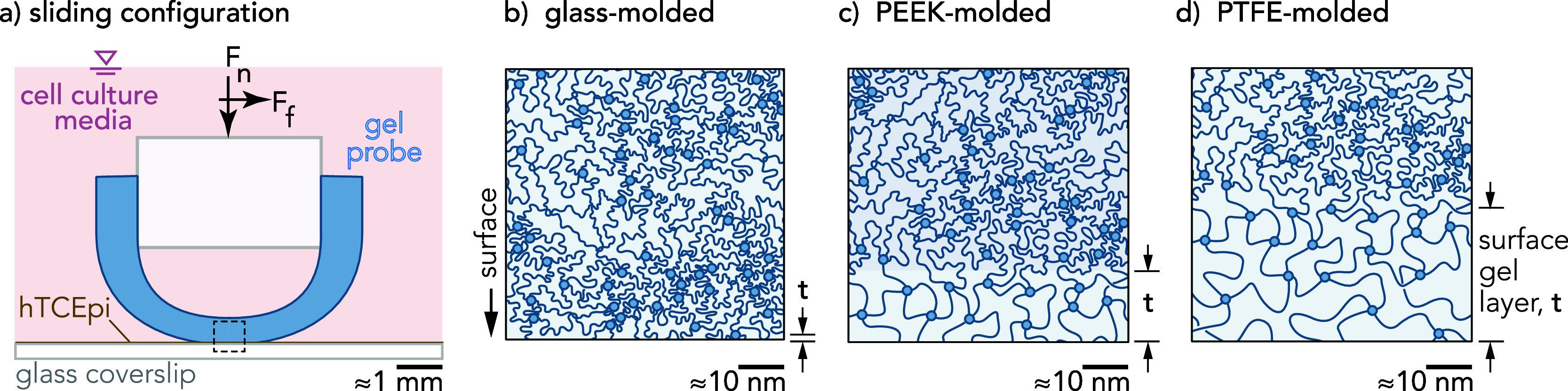
(a) Side view schematic of hydrogel shell probes in sliding contact with hTCEpi cell monolayers submerged in cell culture media. (b–d) Hypothesized surface gel layer thickness, *t*, following hydrogel polymerization in three different mold materials: glass, PEEK, and PTFE.

**Figure 2:**
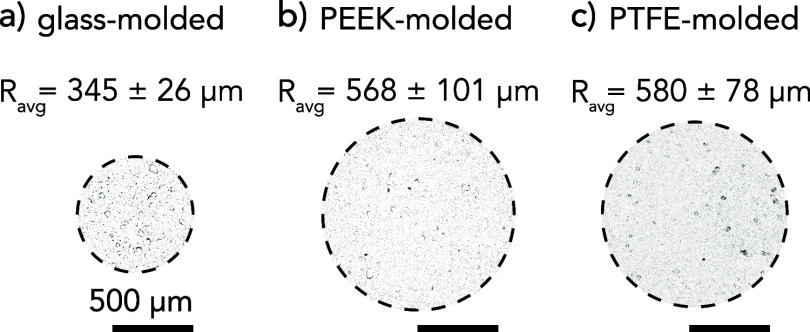
Hydrogel probes polymerized in (a) glass molds, (b) PEEK molds, and (c) PTFE molds distribute the same normal force (*F*_n_ ≈ 250 μN) across different apparent areas of contact against cell layers: *A_glass_*≈ 0.37, *A_PEEK_* ≈ 1.02, and*A*_PTFE_ ≈ 1.07 mm^2^. Probe contact area measurements were made using in situ confocal microscopy imaging during which cell deformation was measured under a maximum normal load of *F*_n_ = 250 μN.

Cell friction measurements were conducted by using a custom-built microtribometer with integrated confocal microscopy. The microtribometer (described in Urueña et al.^60^) was mounted in the condenser turret of a Nikon A1R HD Ti2 confocal inverted microscope such that the hydrogel probe was centered along the optical path. Normal and friction forces were measured using a titanium double-leaf cantilever flexure assembly (normal stiffness: *K*_n_ = 200 μN μm^–1^, tangential stiffness: *K*_t_ = 125 μN μm^–1^) with capacitance probes mounted in the normal and tangential directions (Lion Precision, model no. CPL190, 5 μm V^–1^ sensitivity, 200 μm range). Cells were placed in a custom-built incubator on the microscope platform. Hydrogel probes (Figure 1) were mounted to the cantilever and lowered using manual micrometer stages for coarse motion and brought into contact with cells using a piezoelectric stage (Physik Instrumente, model no. E-01.621) and loaded to a normal force of *F*_n_ ≈ 250 μN. The motorized microscope stage provided constant velocity (*v* = 1 mm/s) across reciprocating motions (*l* = 3 mm). Fluorescent images (4 and 20× magnification) of hydrogel probes in contact with cell layers were used to calculate the contact area (Figure 2). Composite fluorescent images of cells within the entire sliding path were acquired before and after tribological experimentation to quantify mucin gel intensity (Supporting Information Section 3).

## Results and Discussion

### Contact Area Measurements

Contact area was evaluated for hydrogel probes in stationary contact with cell monolayers between 80 and 90% confluence. Measurements of the contact area were taken prior to tribological testing using a confocal microtribometer with fluorescence microscopy over a single imaging plane. Pristine hydrogel probes polymerized in custom PTFE, PEEK, and glass molds and equilibrated in PBS were loaded into contact with fluorescently labeled cells to a maximum normal force of *F*_n_ ≈ 250 μN. The contact area was determined from the region of deformed cells that appeared in the field of view at 4× magnification. Image differencing methods^61^ were used to determine cell and mucin gel deformation during contact. These results were inverted to estimate the boundaries of contact across cell monolayers. The contact diameters of these regions determined the apparent contact areas (Figure 2), which were divided by the normal force to estimate the average contact pressure: *P*_PTFE_ ≈ 210 (*N* = 3), *P*_PEEK_ ≈ 270 (*N* = 6), and *P*_glass_ ≈ 690 Pa (*N* = 3).

### Nanoindentation Measurements

The mechanical properties of hydrogel surfaces polymerized against PTFE, PEEK, and glass were characterized using nanoindentation (Optics11 Life Pavone, NSF BioPACIFIC Materials Innovation Platform at UC Santa Barbara). The nanoindenter was fit with a smooth silica colloidal probe (Optics11 Life Well Plate Probe, cantilever stiffness, *K*_nano_ = 0.025 N m^–1^, tip radius, *R*_nano_ = 25 μm for PTFE-, PEEK-molded gels; Optics11 Life Well Plate Probe, cantilever stiffness, *K*_nano_ = 0.47 N m^–1^, tip radius, *R*_nano_ = 25 μm for glass-molded gels) and used to evaluate the reduced elastic modulus of each hydrogel sample (*N* = 2 independent samples, *n* = 3 locations per sample) using either the Hertzian (eq 1) or Winkler (eq 2) contact mechanics models. In both the Hertz and Winkler contact mechanics models, the applied normal force, *F*_n_, is a function of E*, the reduced elastic modulus, *R,* the radius of curvature of the probe, and *d*, the indentation depth. The Winkler foundation model also depends on *t*, which is the approximate thickness of the surface gel layer. In this work, *t* was estimated to be 100 μm, which was informed by previous work by our group and others.^44,48^
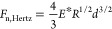
1
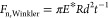
2

**Figure 3:**
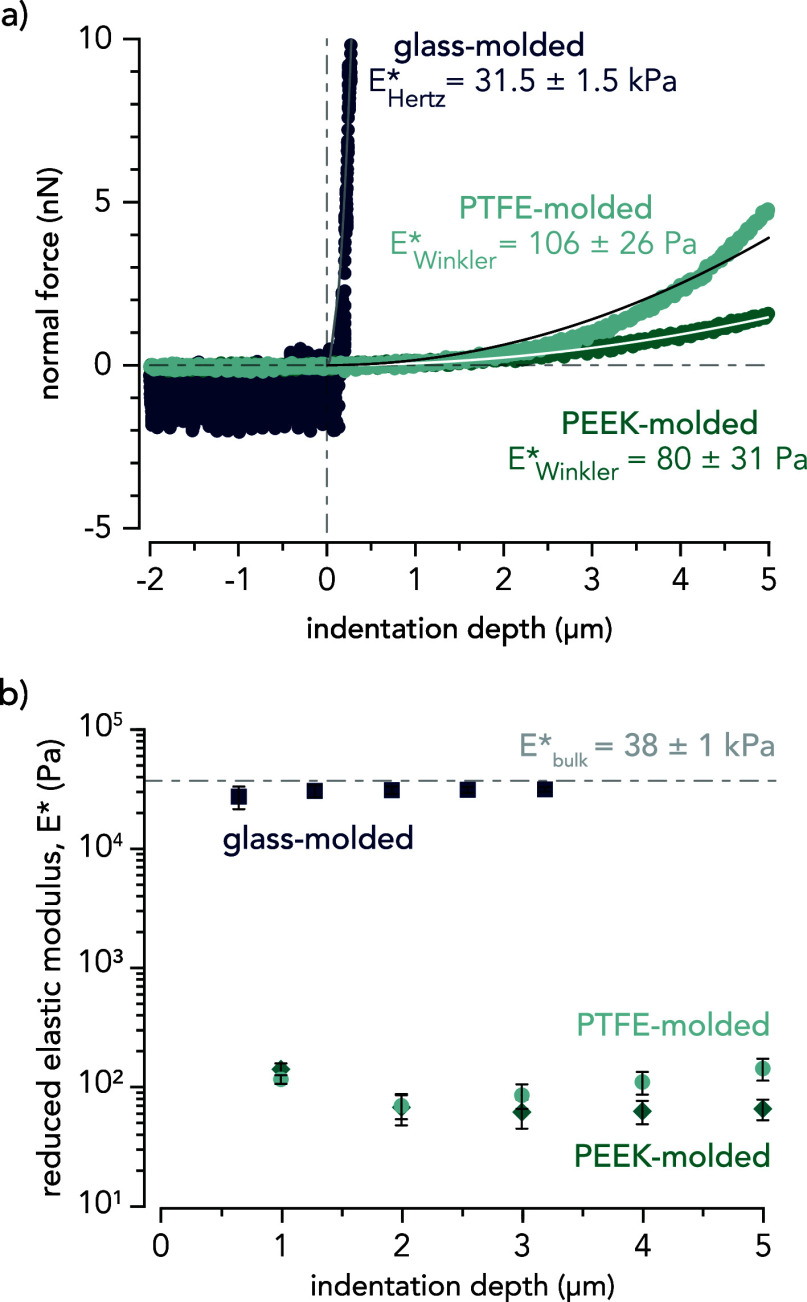
Nanoindentation measurements of glass-, PEEK-, and PTFE-molded hydrogel surfaces against spherical glass probes. (a) Approach curves were fit by using the Hertzian contact mechanics model for glass-molded gels (gray line) and the Winkler contact mechanics model for the PEEK- and PTFE-molded gels (white and black lines, respectively). These fits were used to determine the reduced elastic modulus, E*, at the surface. (b) The reduced elastic modulus was determined by fitting the nanoindentation data at varying depths using the Hertz contact mechanics model (glass-molded) and the Winkler contact mechanics model (PTFE- and PEEK-molded). *N* = 2 independent samples, *n* = 3 locations per sample. Error bars in the figure represent ±1 standard deviation.

The Winkler model exhibited the closest fits to the experimental data for hydrogels cast against PEEK and PTFE, while the Hertz model provided the closest fits for gels cast against glass (Supporting Information Section 5). The Winkler model or ‘bed-of-springs foundation model’ resulted in closer fits to the data for the PTFE- and PEEK-molded hydrogel surfaces due to their surface gel layer, which, under these loading conditions, act as a layer of parallel springs. The loosely cross-linked polymer network of the surface gel layer can be likened to a “brushy” surface, as often described by Spencer and co-workers.^38,39^ Gel samples were indented to a maximum depth of ≈3 μm for glass-molded gels and ≈5 μm for PTFE- and PEEK-molded gels. Indentation data from these measurements were fit to multiple indentation depths, *d*, using Hertzian and Winkler contact mechanics models. These models were used to estimate the reduced elastic modulus, E*, as a function of depth (Figure 3). The reduced elastic modulus of glass-molded gels at an indentation depth of *d* ≈ 640 nm was  = 28,518 ± 6,100 Pa. Indenting further into the sample (*d* ≈ 3,180 nm) resulted in  = 32,810 ± 1,470 Pa. Although a thin surface gel layer on the glass-molded hydrogel was detected and estimated to be less than *t* < 1 μm, the mechanical properties of the bulk hydrogel network dominated ( = 37,915 ± 1,000 Pa) which necessitated the use of the Hertzian contact mechanics model for all indentation depths. The average and standard deviation of the reduced elastic moduli for PTFE-, PEEK-, and glass-molded hydrogel surfaces at maximum indentation depths (*d*_max,PTFE_ = *d*_max,PEEK_ = 5.00 μm and *d*_max,glass_ = 3.18 μm) are  = 106 ± 26,  = 80 ± 31, and  = 31,560 ± 1,570 Pa, respectively. The manufacturer-specified lower limit of elastic modulus measurements for the Optics11Life Pavone nanoindenter under these conditions was E* = 10 Pa.

### Biotribological Measurements

**Figure 4:**
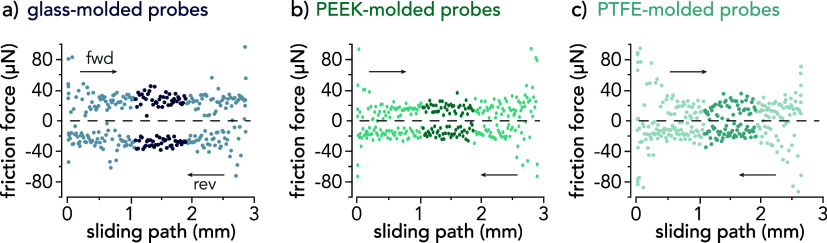
Representative friction force traces vs position for one reciprocating cycle for (a) glass-molded, (b) PEEK-molded, and (c) PTFE-molded hydrogel probes sliding against hTCEpi cell monolayers. The free sliding regime (middle 20% of the sliding path) is indicated by the darker data points. Friction force traces were randomly selected within the last 200 cycles (steady-state regime) and correspond with cycles 507, 421, and 576 for sliding experiments conducted with glass-, PEEK-, and PTFE-molded probes, respectively. The average normal force over the highlighted regions for each experiment is *F*_n_ ≈ 250 μN.

Six independent tribological experiments (*N* = 6) were conducted for each hydrogel probe (PTFE-, PEEK-, and glass-molded) against hTCEpi monolayers grown on separate glass-bottom culture dishes. All sliding experiments were conducted with probes and cell layers fully submerged in cell culture media. Each experiment was conducted in normal cell culture growth conditions for 600 reciprocating cycles (3.6 m total sliding distance) under a steady normal force of *F*_n_ ≈ 250 μN and a constant sliding velocity of *v* = 1 mm/s. The duration of each sliding experiment was about 1.5 h and imaging before and after required an additional 30 min. Representative friction force traces from the last 200 sliding cycles (steady-state regime) are shown in Figure 4. Some breakloose friction or static friction is observed at the reversals (Supporting Information Section 6).

**Figure 5:**
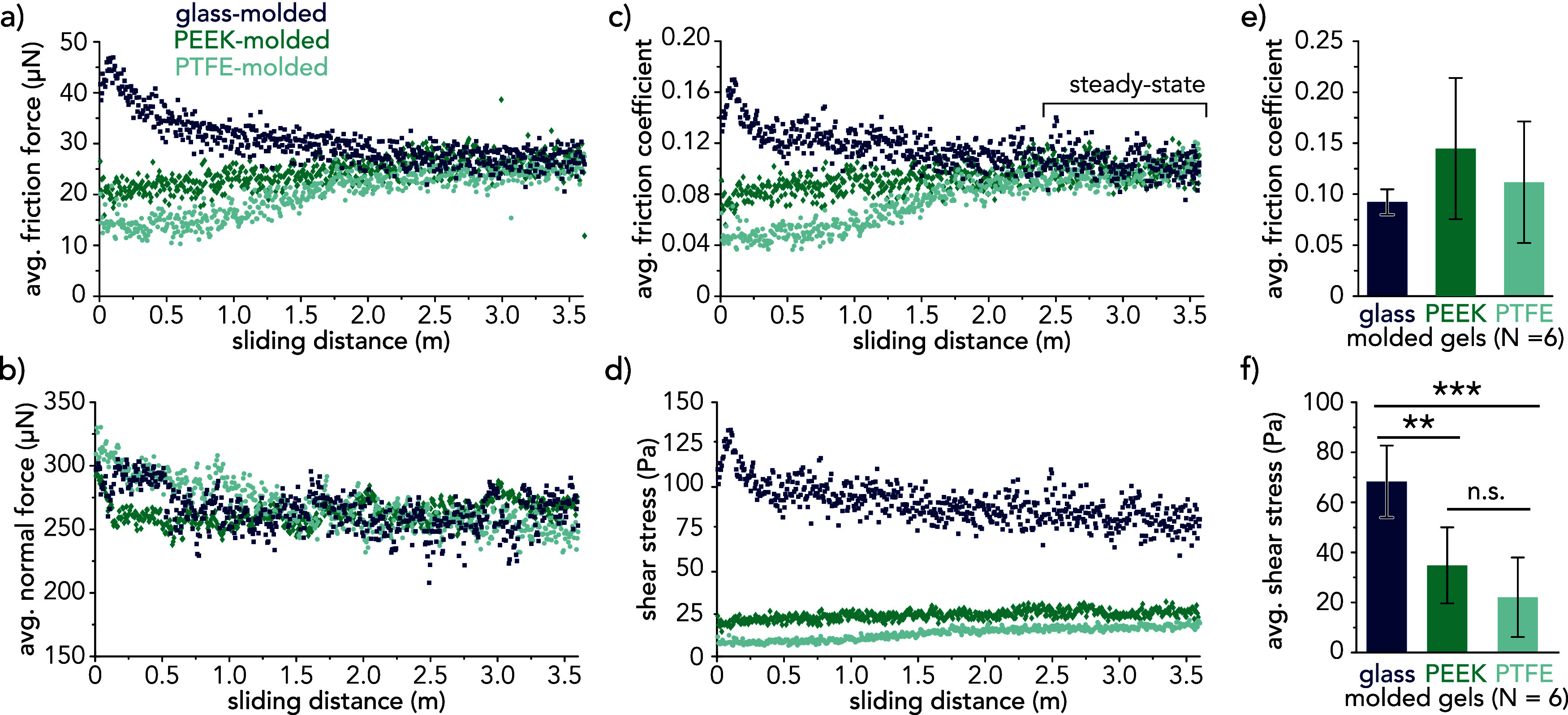
Representative data for 600 cycles (3.6 m sliding distance) for hydrogel probes sliding against hTCEpi cell monolayers: (a) average friction force per cycle, (b) average normal force per cycle, (c) average friction coefficient per cycle, and (d) average shear stress per cycle. Data are shown for three types of hydrogel probes: glass-molded (blue), PEEK-molded (green), and PTFE-molded (light green). (e) Bar plot showing the average free-sliding friction coefficient calculated from the steady-state regime for glass-molded, PEEK-molded, and PTFE-molded hydrogel probes (*N* = 6 per mold type). No statistical significance exists between the friction coefficients for tested gel samples. (f) Bar plot showing the average free-sliding frictional shear stress for glass-molded, PEEK-molded, and PTFE-molded hydrogel probes (*N* = 6 per mold type). Student’s *t* test showed statistically significant differences between glass-molded and PEEK-molded (*p* = 0.0033) and glass-molded and PTFE-molded (*p* = 0.00020) but not PEEK-molded and PTFE-molded (*p* = 0.0761). Averages from all experiments were determined using the steady-state regime (indicated above). Error bars represent ±1 standard deviation.

The average friction force within the free sliding regime per cycle is shown as a function of sliding distance for each of the hydrogel probes in Figure 5. Over the first 0.5 m of cumulative sliding distance against hTCEpi cell monolayers, the average friction force was the lowest for PTFE-molded probes (*F*_f,avg_ ≈ 13 μN), followed by PEEK-molded probes (*F*_f,avg_ ≈ 22 μN) and then by glass-molded probes (*F*_f,avg_ ≈ 40 μN). After approximately 2 m of total sliding distance, the average friction forces for the three hydrogel probes reached a “steady-state” regime marked by relatively consistent friction forces (*F*_f,steady_ ≈ 26 μN) for the remainder of the sliding experiment. The increase in frictional forces observed within the initial 2 m sliding distance for probes with surface gel layers may indicate the local collapse of the surface gel layer. Nanoindentation measurements (Figure 3) show that the reduced elastic modulus, E*, at the PTFE- and PEEK-molded surfaces are 2–3× lower than the maximum applied contact pressures (*P*_PTFE_ ≈ 210 Pa, *P*_PEEK_ ≈ 270 Pa). However, this was not the case for the glass-molded gels (*P*_glass_ ≈ 690 Pa). For hydrogels cast against PTFE and PEEK, it is possible that the mesh size at the surface could be decreasing over the course of the experiment due to local draining from persistently high contact pressures^62^ relative to the polymer osmotic pressure. An additional consideration is the accumulation of protein such as cellular debris, which remains adhered to all probes at the conclusion of each experiment (Supporting Information Section 7). Future investigations will focus on the role of adsorbed polymers and proteins in the mechanical and tribological properties of hydrogel surfaces.

Average friction coefficients were calculated from the last 200 reciprocating cycles (2.4 to 3.6 m sliding distance) for each of the hydrogel probes. The average friction coefficient results from six independent sliding experiments (*N* = 6) are plotted in Figure 5e. The average friction coefficient and standard deviation for each of the hydrogel probes over 200 cycles across six independent experiments are μ_glass_ = 0.092 ± 0.012, μ_PEEK_ = 0.145 ± 0.069, and μ_PTFE_ = 0.112 ± 0.060. For each of the hydrogel probes, the average frictional shear stress (Figure 5f) was calculated from the product of the average friction coefficient and the average contact pressure, which was determined from indentation measurements against hTCEpi cell layers: τ_glass_ = 68.2 ± 14.5, τ_PEEK_ = 35.2 ± 15.3, and τ_PTFE_ = 22.4 ± 16.0 Pa. It is noteworthy that hydrogel probes with surface gel layers exhibited frictional shear stresses within the free sliding regime well below the critical threshold correlated with the initiation of apoptosis in hTCEpi cell monolayers (τ ≈ 80 Pa).^50^

Although the average friction coefficients for PEEK-, PTFE-, and glass-molded probes were similar, the average frictional shear stresses revealed statistically significant differences (Figure 5f). As the concept of “lubricity” is often defined as the product of low friction coefficient and low contact pressure,^12,32,63^ the PTFE- and PEEK-molded probes, owing to their greater compliance (i.e., greater contact areas, Figure 2) compared to the glass-molded probe, exhibit lower frictional shear stresses and thus are considered more “lubricious”.

**Figure 6:**
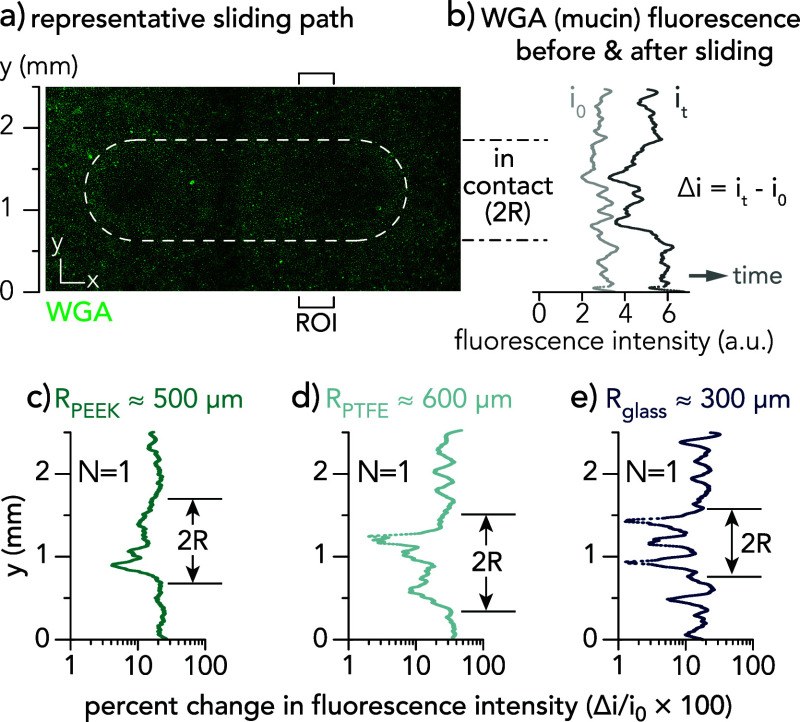
(a) A representative sliding path (indicated by a white dotted line) over a cell monolayer is shown. The region of interest (ROI) is a sample area measuring 450 μm by 2,500 μm, spanning the free sliding regime and regions out of contact, and is used to analyze the fluorescent intensity of wheat germ agglutinin (WGA), a proxy for mucin intensity. (b) The pixel intensity values across the ROI are shown both before and after a sliding experiment over the cell monolayer. The change in pixel intensity relative to the initial intensity is depicted for (c) PEEK-molded, (d) PTFE-molded, and (e) glass-molded probes.

Outside of the sliding path, the mucin pixel intensity from WGA staining increased by about 30%, which indicates continuous mucin production throughout the experiment. Analyzing the mucin pixel intensity before and after tribological testing within the sliding path reveals that intricate mucin networks can be disrupted at frictional shear stresses as low as τ ≈ 11 Pa. Moreover, the distance over which this change in intensity occurs corresponds to the contact diameter of the probe (Figure 6) which was previously measured from indentation (Figure 2). The confocal microscope settings (e.g., laser power) were maintained for all investigations of mucin intensity before and after sliding.

**Figure 7:**
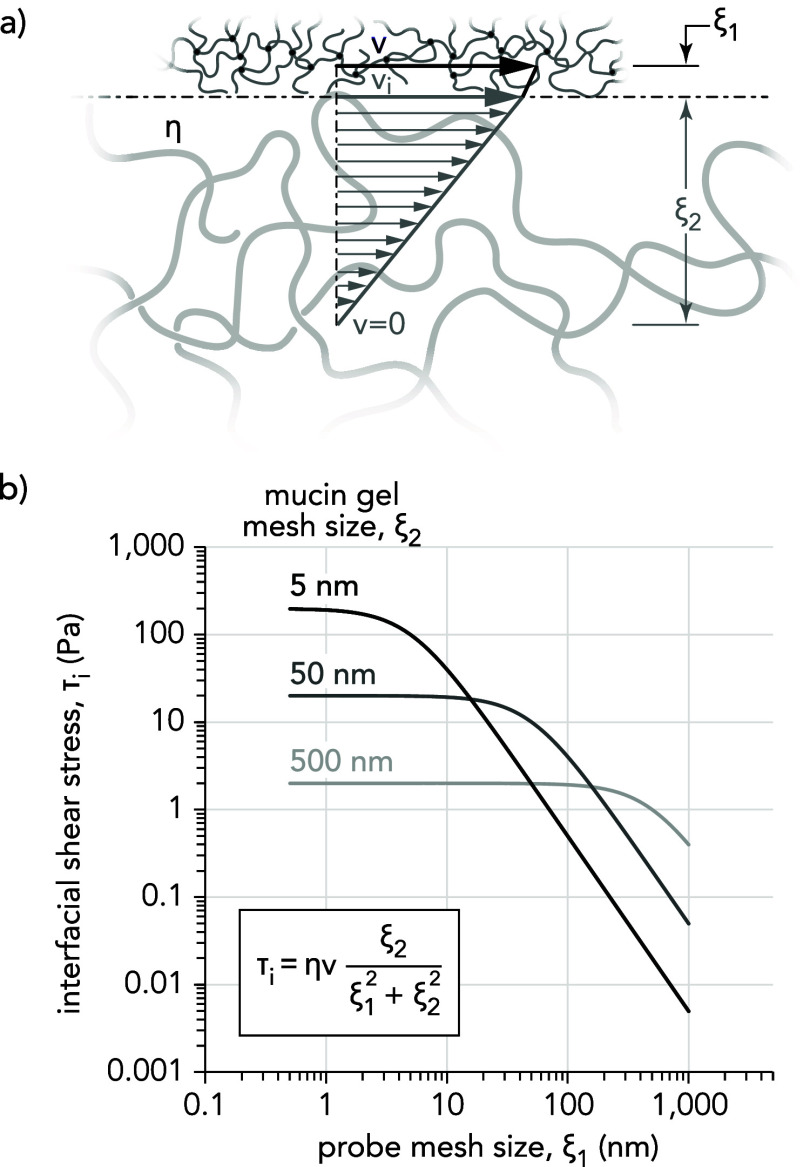
(a) An illustration of the fluidized shear that occurs at a mixed mesh size sliding interface. (b) A proposed model depicting the change in interfacial shear stress as a function of both the surface polymer mesh size of the probe (ξ_1_) and the mucin gel mesh size (ξ_2_). Adapted from ref (64).

The mesh size of the gel-spanning network of the tear film is often reported to be between 100 and 200 nm,^65^ a length scale which effectively bars the entry of pathogens and debris. Such a large mesh size may also maintain robust lubrication despite tribological challenges including contact lens wear. The buried interface between the surface of a contact lens sliding against the cornea could be approximated as a mixed mesh size interface (Figure 7a).^64^ In the mixed interface model, a tribological pair of two different mesh sizes above (ξ_1_ ≥ 0) and below (ξ_2_ > 0) the sliding interface share an equivalent fluid shear stress, τ_i_. The top polymer network slides at a velocity of *v* and the velocity of the interface is given by *v*_*i*_. The fluid velocity gradient is assumed to have a hydrodynamic penetration depth of one mesh size into each of the tribological pairs.^66^ At thermal fluctuation lubrication sliding speeds (*v* = 1 mm/s), the system is assumed to be shear-thinning, and thus the viscosity, η, scales inversely with mesh size. The interfacial shear stress, τ_i_, can be written as follows:
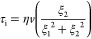
3

Equating ξ_1_ = ξ_2_= ξ gives an expression for interfacial shear stress that is similar to that of shear stress in a matched mesh size (Gemini) interface.^63^ While the exact mesh size of the mucin gel network and the surface of the hydrogel probes are unknown, Figure 7b depicts the estimated fluid shear stress, τ_i_, across the mixed interface for a range of hydrogel probe mesh sizes and for three different mesh sizes of the mucin gel network: ξ = 5, 50, and 500 nm. The model suggests that the shear stress of the sliding interface is dominated by the polymer network with larger mesh size. This may explain why three different types of hydrogel surfaces in sliding contact with mucin-producing cell monolayers exhibited similar friction coefficients—the mesh size of the mucin gel network is always larger. However, as the mucin gel layer is either compressed or damaged throughout the experiment, the friction coefficient and thus the frictional shear stress may depend more strongly upon the larger mesh size of the hydrogel probe for energy dissipation.

## Limitations

The presence of the surface gel layer can frustrate efforts to produce flawless probes without defects (e.g., holes). This may be due to the fact that the backing post, composed of polyoxymethylene (POM), has a surface tension similar to that of PEEK and may create a surface gel layer on both sides of the hydrogel probe. This has the added effect of creating greater compliance (larger contact area), but it also leads to more probes that are too thin and fracture upon removal from the probe mold. Oxygen may also increase the variability in the surface gel layer thickness from probe to probe. All solutions are prepared under ambient conditions without displacing oxygen and without the use of inert atmospheres during polymerization. These decisions were deliberate in order to simulate the large-scale operations commonly found in industrial settings. The use of glove boxes to maintain an inert environment likely increases the cost of production and lowers throughput for commercially available products, such as contact lenses. Therefore, the use of widely available polymer chemistry that is well-characterized and amenable to real-world industrial conditions is a feature of this approach.

Another limitation of this study is that this investigation uses hTCEpi cells, which are known to produce only membrane-bound mucins. The addition of gel-forming mucins, such as MUC2, presents its own set of challenges, including difficulties associated with purification. However, this investigation could provide insights into the ability of surface gel layers to protect vulnerable, mucin-deficient ocular surfaces in the cases of dry eye disease, Sjögren’s syndrome, or in harsh environments.

## Conclusions

The “mold effect” first observed by Gong et al.^40^ and demonstrated by Spencer and co-workers to be a function of oxygen-inhibition polymerization^37−39^ was leveraged to create surface gel layers on hydrogels that mimic gradient mucin gels across biological sliding interfaces. In this investigation, surface gel layers were created by polymerizing polyacrylamide hydrogels (7.5 wt % acrylamide, 0.3 wt % *N,N'*-methylenebisacrylamide) within PEEK and PTFE hemispherical molds. Nanoindentation measurements determined that hydrogels polymerized within glass molds exhibited significantly larger reduced elastic modulus at the surface ( = 31,560 ± 1,570 Pa) compared to those cast in PEEK ( = 80 ± 31 Pa) or PTFE molds ( = 106 ± 26 Pa). In vitro investigations indicate that surface gel layers protect delicate hTCEpi cell layers by reducing frictional shear stresses during sliding contact (*τ*_PEEK_ = 35 ± 15 and *τ*_PTFE_ = 22 ± 16 Pa) compared to hydrogels with higher polymer density at the surface (*τ*_glass_ = 68 ± 15 Pa). The relative abundance of mucin gels on the surfaces of hTCEpi cells before and after sliding (applied normal force, *F*_n_ ≈ 250 μN; sliding speed, *v* = 1 mm/s, stroke length, *l* = 3 mm, total sliding distance, *d* = 3.6 m) provide further evidence that surface gel layers protect hTCEpi cells and can improve the biocompatibility of device-tissue sliding interfaces. Future studies will investigate the extent to which the surface gel layer of hydrogel probes is influenced and controlled by surface treatments, mold material, and surface roughness. Further investigations will explore the role of mucin gel removal following sliding experiments on the gene expression profiles of hTCEpi cells and the impact of dwell on breakloose friction.

## Data Availability

Data is freely available at Dryad: https://doi.org/10.5061/dryad.c2fqz61hs.

## Supporting Information Available

Microscopy data of gel probe cross sections (Section 1), immunofluorescence staining of mucin (Section 2), composite images of cell sliding experiments (Section 3), cell death quantification data (Section 4), indentation data for gel surface characterization (Section 5), analysis of breakloose friction (Section 6), mucoadhesion to gel probes (Section 7) (PDF)
