# Supplementary material for: Bioinspired Lubricity from Surface Gel Layers
Source: Langmuir. 2024 Apr 29;40(19):9926–33. doi: 10.1021/acs.langmuir.3c03686 (PMC11100014; doi:10.1021/acs.langmuir.3c03686)
Supplement: Supplementary file 1 — la3c03686_si_001.pdf [file la3c03686_si_001.pdf]

Supporting Information

Bioinspired Lubricity from Surface Gel Layers

Ahmed Al Kindi[a], Nemea S. Courelli[b], Kevin Ogbonna[c], Juan Manuel Urueña[d], Allison L. Chau[e], and Angela A. Pitenis[e*]

[a]Department of Mechanical Engineering, University of California, Santa Barbara, Santa Barbara, CA 93106
[b]Department of Chemical Engineering, University of California, Santa Barbara, Santa Barbara, CA 93106
[c]College of Creative Studies, Biological Sciences, University of California, Santa Barbara, Santa Barbara, CA 93106
[d]NSF BioPACIFIC Materials Innovation Platform, University of California, Santa Barbara, Santa Barbara, CA 93106
[e]Materials Department, University of California, Santa Barbara, Santa Barbara, CA 93106

*Corresponding author
Angela A. Pitenis
apitenis@ucsb.edu

Number of pages: 60
Number of figures: 46
Number of tables: 1

Section 1: The membrane thickness for hydrogels probes cast against glass (N=5), PEEK (N=4) and PTFE (N=5) was measured using fluorescent confocal microscopy. No statistically-significant difference was found between the membrane thicknesses of the probes used in sliding experiments. (p-values for each experiment: $p_{glass - PEEK} = 0.18$, $p_{glass – PTFE} = 0.51$, $p_{PEEK – PTFE} = 0.54$)

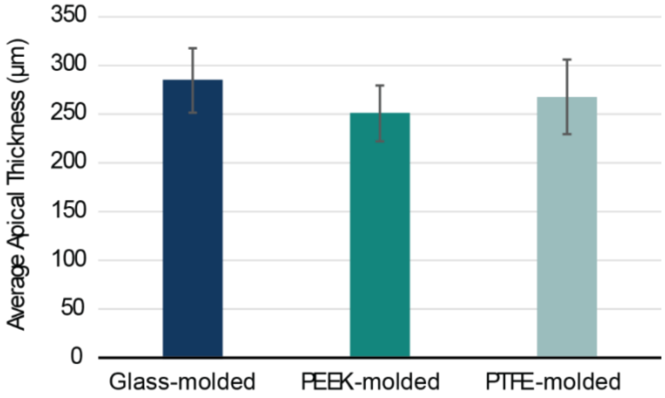

Figure S1. Average apical thickness of the spherical shell probes molded against glass, PEEK, and PTFE measured by confocal fluorescence microscopy. Error bars represent ±1 standard deviation.

Section 2: Immunofluorescent antibody stains of fixed human telomerase-immortalized corneal epithelial (hTCEpi) cells showing membrane-bound mucin network.

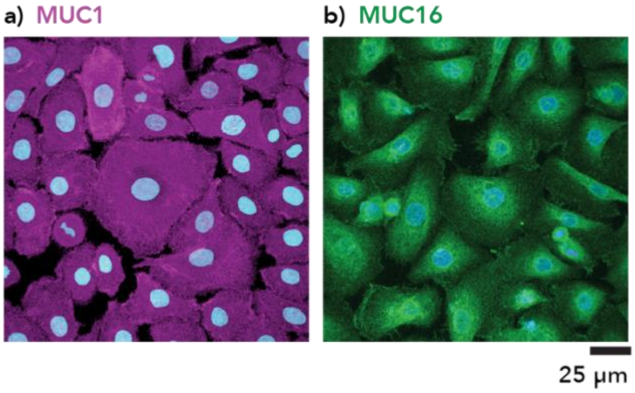

Figure S2. Human telomerase-immortalized corneal epithelial (hTCEpi) cells fixed and stained for (a) MUC1 (magenta) and (b) MUC16 (green). Cell nuclei are stained blue (Hoechst 33342).

Section 3: Composite fluorescent images of 6 independent sliding paths before and after sliding for A) PEEK-molded, B) PTFE-molded, and C) glass-molded probes, respectively, against hTCEpi cell monolayers.

Note: For all images in this section, mucin (red) is stained with wheat germ agglutinin (conc. 0.085 μM) and cell bodies (green) are stained with CellTracker™ Green CMFDA Dye (conc. 5 μM). Composite images were taken once before sliding and once after sliding.

3A. Sliding experiments using PEEK-molded gel probes.

<u>Experiment 1</u>

Probe: PEEK-molded polyacrylamide hydrogel
Countersurface: hTCEpi cell monolayers
Environment: cell growth media, maintained at 37C, 5% CO_2, >95% relative humidity
Normal force: 250 μN
Contact area ~ 0.95 mm^2
Sliding speed: 1 mm/s
Sliding path length (1/2 cycle) = 3 mm
Total sliding distance: 3.6 m
Number of reciprocating cycles: 600
Duration of sliding experiment: 1 h 21 min
Field of view = 620 μm × 3,000 μm

Objective: 20× (0.62 μm/pixel)
FITC (λ = 488 nm) Laser Power = 0.1; Gain = 14
TRITC (λ = 561 nm) Laser Power = 0.1; Gain = 50
CY5 (λ = 640 nm) Laser Power = 5; Gain = 100

Objective: 4× (3.11 μm/pixel)
FITC (λ = 488 nm) Laser Power = 5; Gain = 15
TRITC (λ = 561 nm) Laser Power = 5; Gain = 50
CY5 (λ = 640 nm) Laser Power = 10; Gain = 100

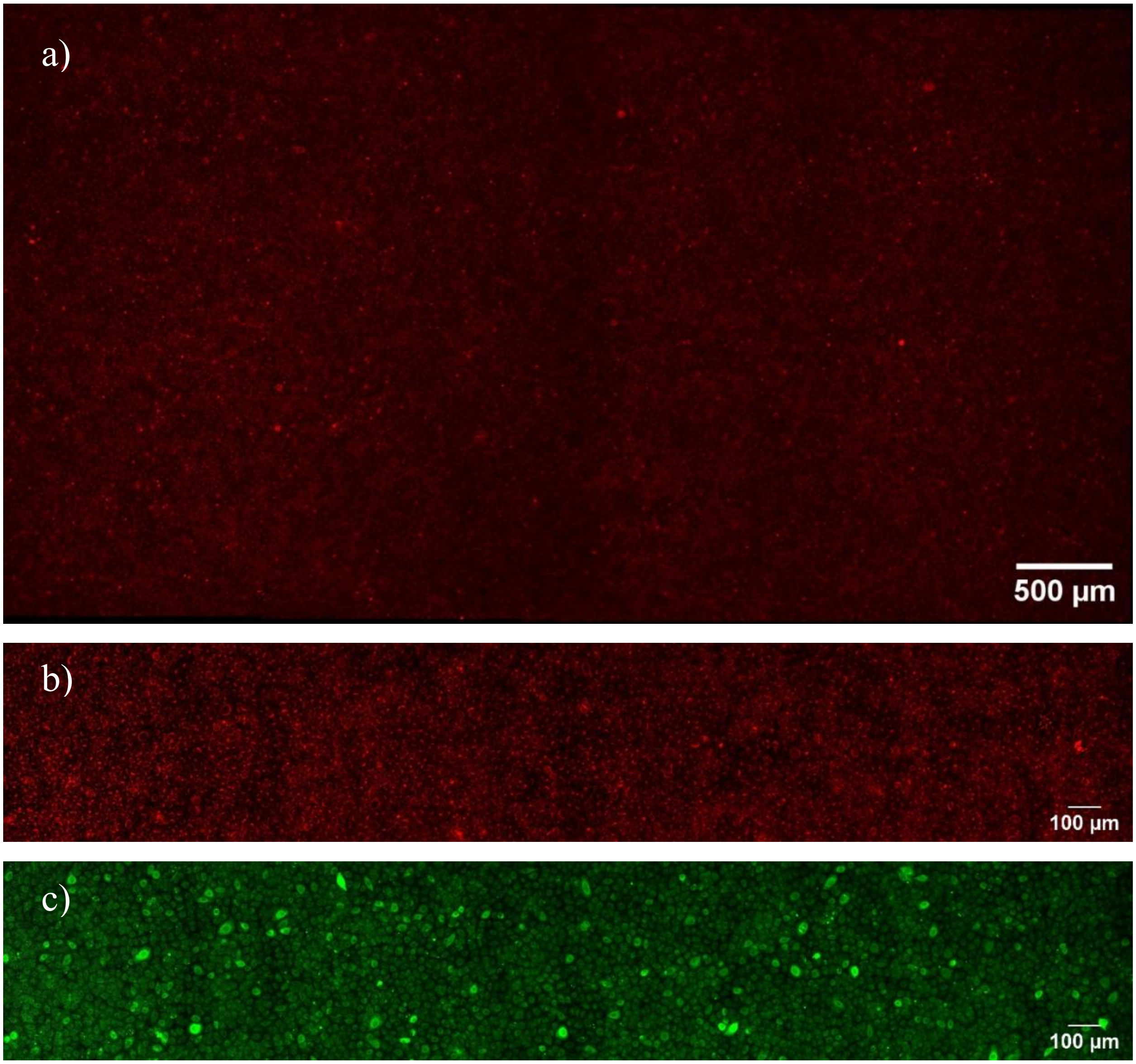

Figure S3. Microscopy images of cells before sliding. a) 4x composite image of cells stained for mucin (red), showing both contacted and non-contacted areas. b) 20x composite image of cells stained for mucin (red) in contact with the probe's sliding path. c) 20x composite image of cells stained with CellTracker™ (green) along the probe's sliding path.

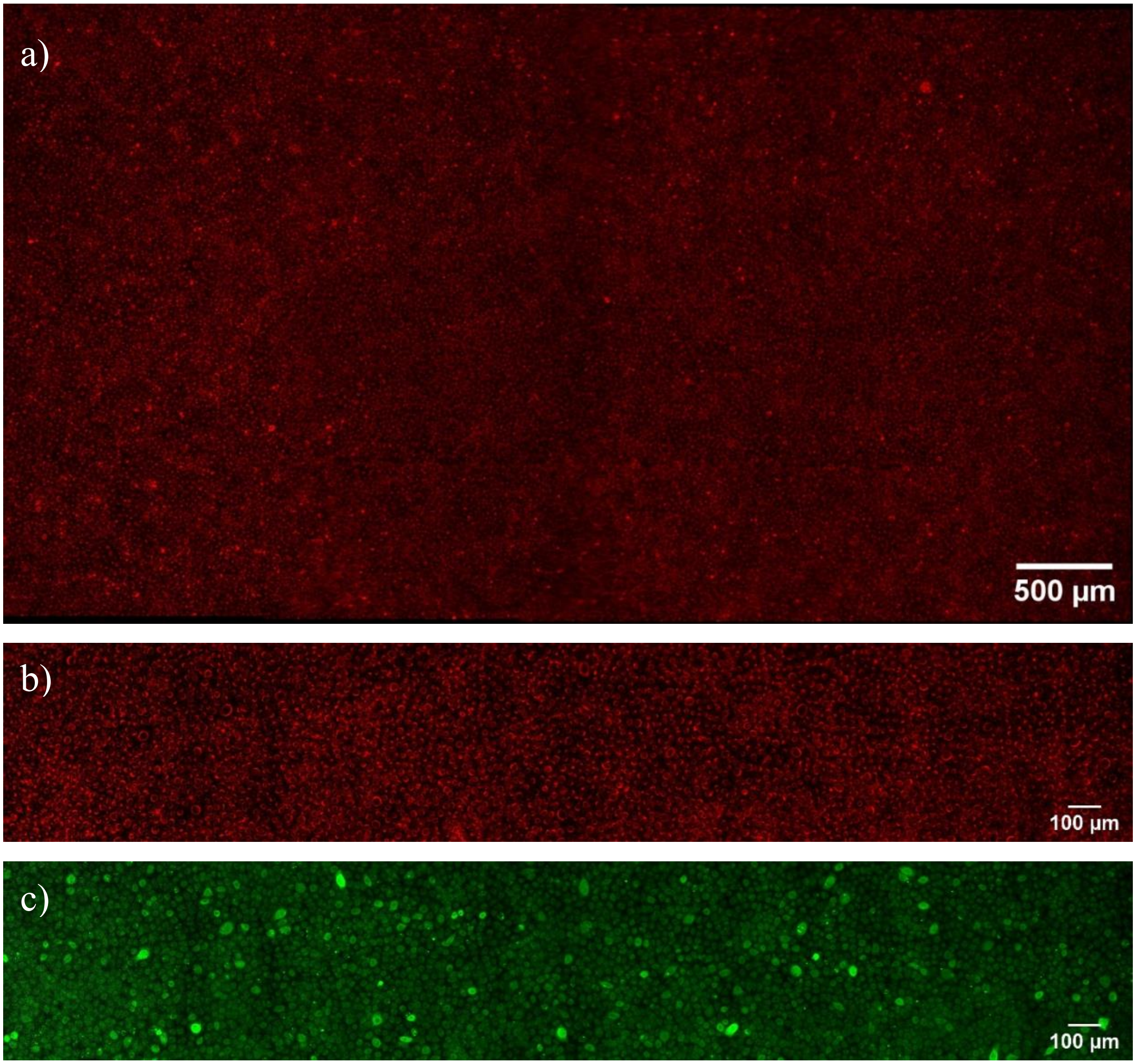

Figure S4. Microscopy images of cells after sliding with a 7.5wt% PAAM gel probe cast against PEEK. a) 4x composite image of cells stained for mucin (red), showing both contacted and non-contacted areas. b) 20x composite image of cells stained for mucin (red) in contact with the probe's sliding path. c) 20x composite image of cells stained with CellTracker™ (green) along the probe's sliding path.

Experiment 2

Probe: PEEK-molded polyacrylamide hydrogel
Countersurface: hTCEpi cell monolayers
Environment: cell growth media, maintained at 37C, 5% CO_2, >95% relative humidity
Normal force: 250 μN
Contact area~ 0.61 mm^2
Sliding speed: 1 mm/s
Sliding path length (1/2 cycle) = 3 mm
Total sliding distance: 3.6 m
Number of reciprocating cycles: 600
Duration of sliding experiment: 1 h 21 min
Field of view = 620 μm × 3,000 μm

Objective: 20× (0.62 μm/pixel)
FITC (λ = 488 nm) Laser Power = 0.1; Gain = 14
TRITC (λ = 561 nm) Laser Power = 0.1; Gain = 50
CY5 (λ = 640 nm) Laser Power = 5; Gain = 100

Objective: 4× (3.11 μm/pixel)
FITC (λ = 488 nm) Laser Power = 5; Gain = 25
TRITC (λ = 561 nm) Laser Power = 5; Gain = 50
CY5 (λ = 640 nm) Laser Power = 10; Gain = 100

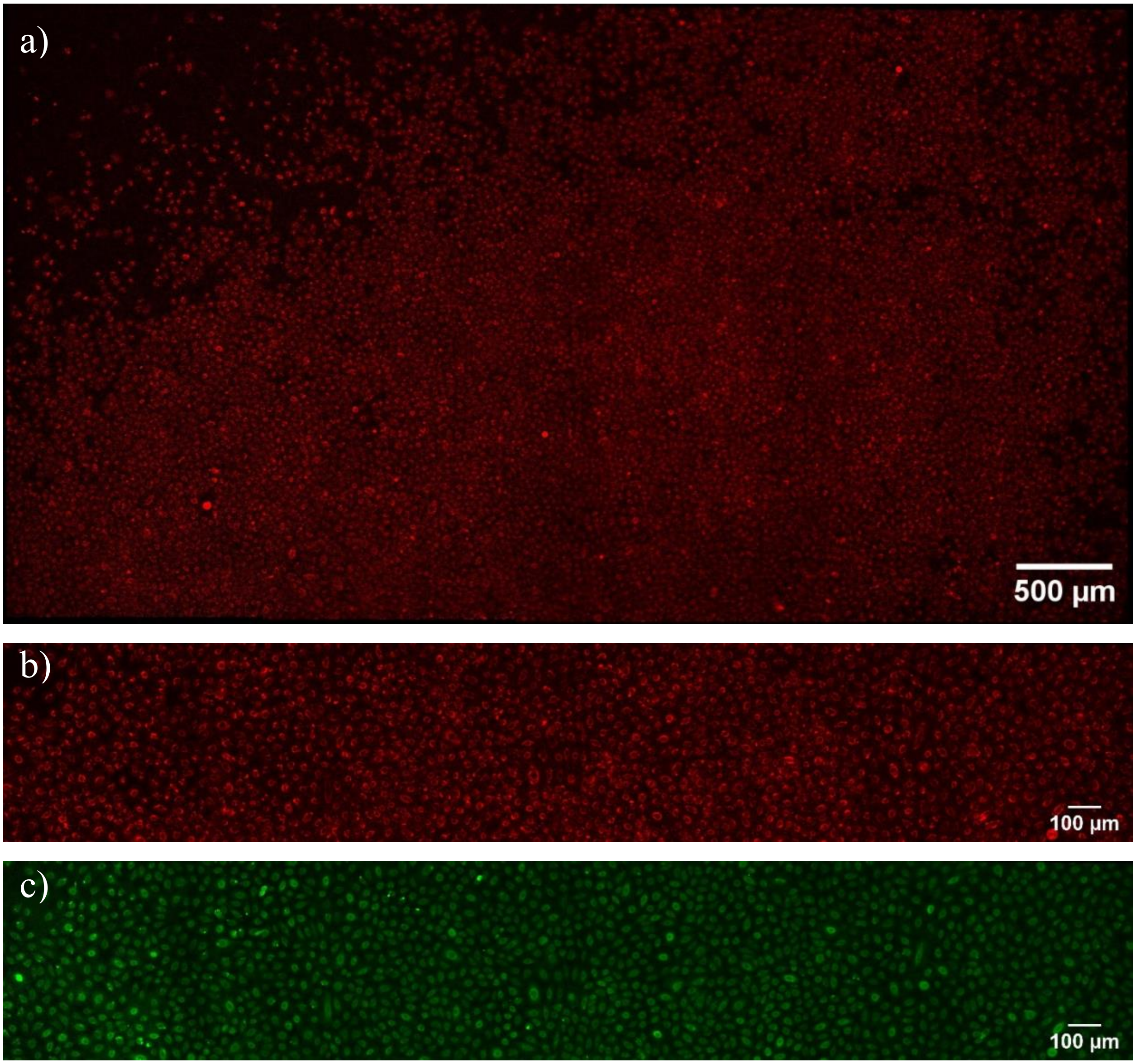

Figure S5. Microscopy images of cells before sliding. a) 4x composite image of cells stained for mucin (red), showing both contacted and non-contacted areas. b) 20x composite image of cells stained for mucin (red) in contact with the probe's sliding path. c) 20x composite image of cells stained with CellTracker™ (green) along the probe's sliding path.

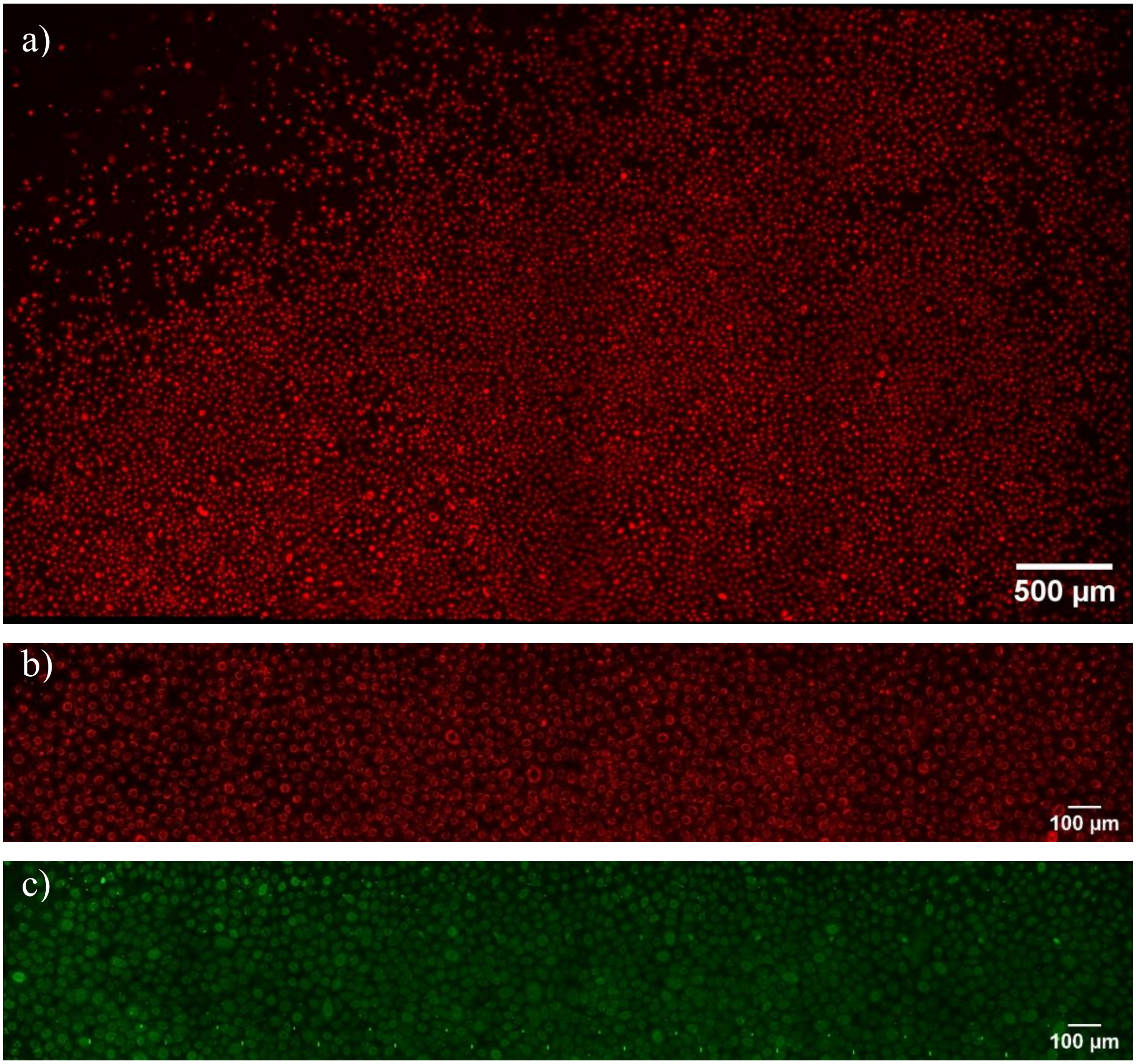

Figure S6. Microscopy images of cells after sliding with a 7.5wt% PAAM gel probe cast against PEEK. a) 4x composite image of cells stained for mucin (red), showing both contacted and non-contacted areas. b) 20x composite image of cells stained for mucin (red) in contact with the probe's sliding path. c) 20x composite image of cells stained with CellTracker™ (green) along the probe's sliding path.

Experiment 3

Probe: PEEK-molded polyacrylamide hydrogel
Countersurface: hTCEpi cell monolayers
Environment: cell growth media, maintained at 37C, 5% CO_2, >95% relative humidity
Normal force: 250 μN
Contact area ~ 1.54 mm^2
Sliding speed: 1 mm/s
Sliding path length (1/2 cycle) = 3 mm
Total sliding distance: 3.6 m
Number of reciprocating cycles: 600
Duration of sliding experiment: 1 h 21 min
Field of view = 620 μm × 3,000 μm

Objective: 20× (0.62 μm/pixel)
FITC (λ = 488 nm) Laser Power = 0.1; Gain = 14
TRITC (λ = 561 nm) Laser Power = 0.1; Gain = 50
CY5 (λ = 640 nm) Laser Power = 5; Gain = 100

Objective: 4× (3.11 μm/pixel)
FITC (λ = 488 nm) Laser Power = 5; Gain = 25
TRITC (λ = 561 nm) Laser Power = 5; Gain = 50
CY5 (λ = 640 nm) Laser Power = 10; Gain = 100

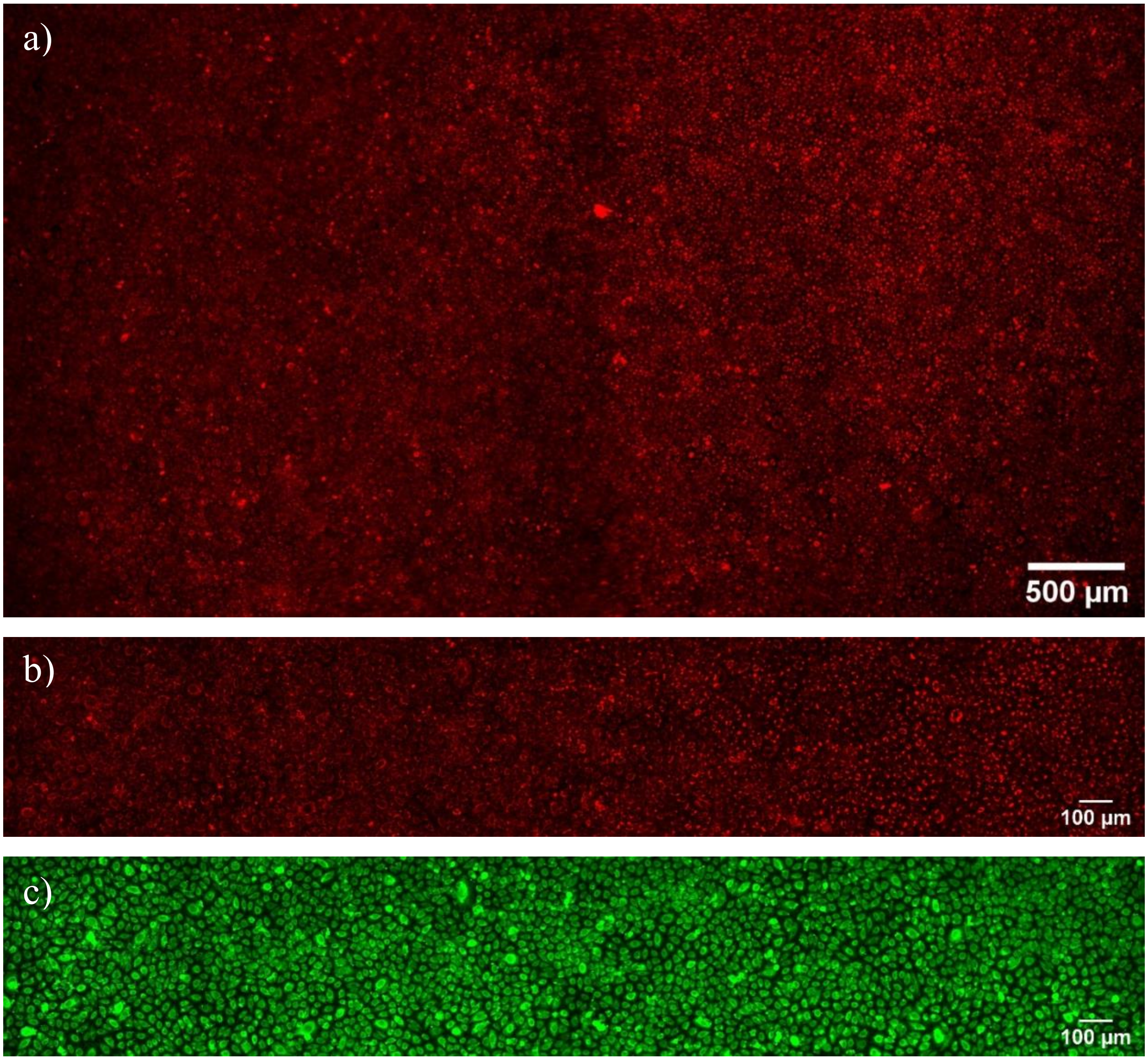

Figure S7. Microscopy images of cells before sliding. a) 4x composite image of cells stained for mucin (red), showing both contacted and non-contacted areas. b) 20x composite image of cells stained for mucin (red) in contact with the probe's sliding path. c) 20x composite image of cells stained with CellTracker™ (green) along the probe's sliding path.

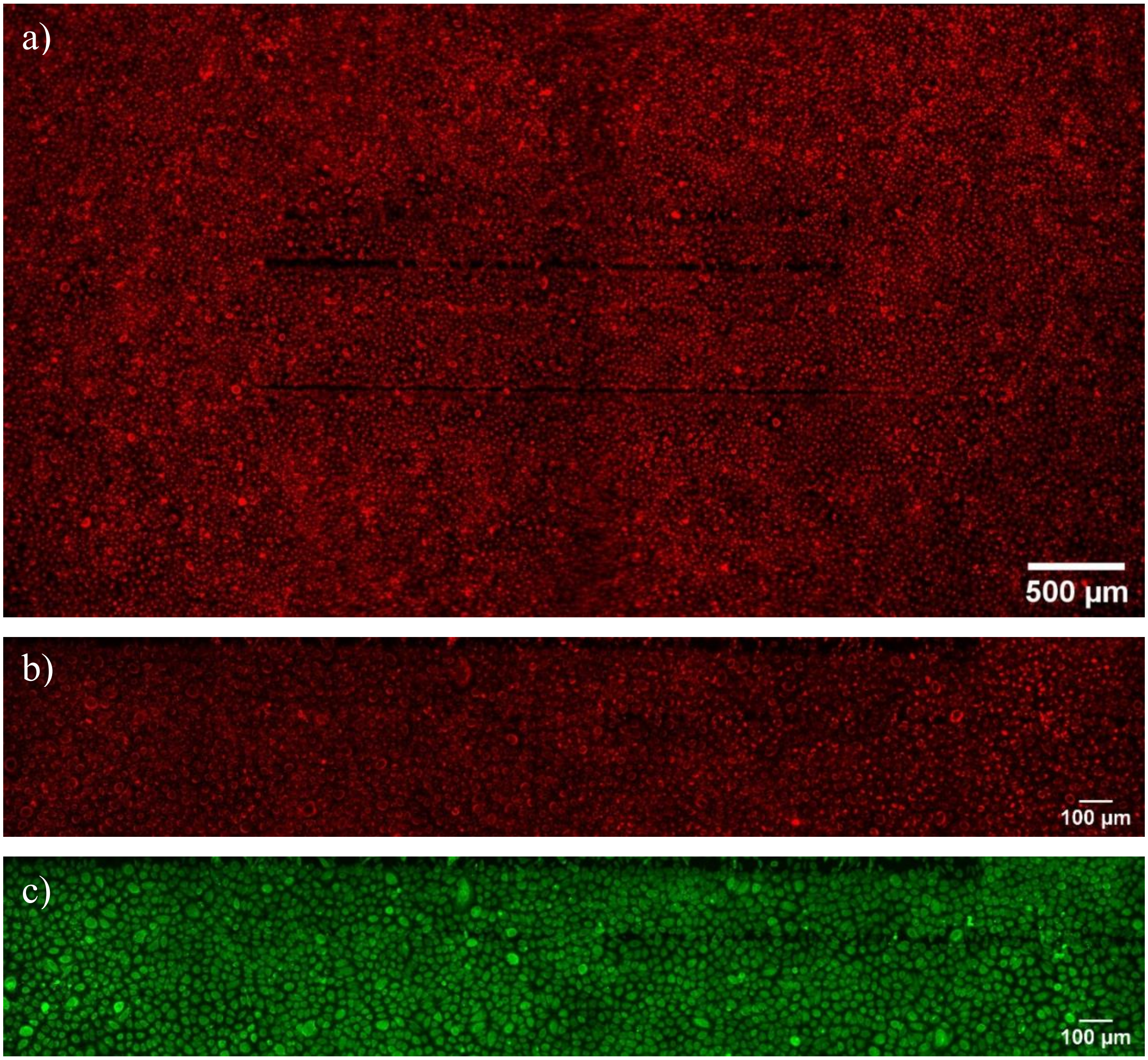

Figure S8. Microscopy images of cells after sliding with a 7.5wt% PAAM gel probe cast against PEEK. a) 4x composite image of cells stained for mucin (red), showing both contacted and non-contacted areas. b) 20x composite image of cells stained for mucin (red) in contact with the probe's sliding path. c) 20x composite image of cells stained with CellTracker™ (green) along the probe's sliding path.

Experiment 4

Probe: PEEK-molded polyacrylamide hydrogel
Countersurface: hTCEpi cell monolayers
Environment: cell growth media, maintained at 37C, 5% CO_2, >95% relative humidity
Normal force: 250 μN
Contact area ~ 1.56 mm^2
Sliding speed: 1 mm/s
Sliding path length (1/2 cycle) = 3 mm
Total sliding distance: 3.6 m
Number of reciprocating cycles: 600
Duration of sliding experiment: 1 h 21 min
Field of view = 620 μm × 3,000 μm

Objective: 20× (0.62 μm/pixel)
FITC (λ = 488 nm) Laser Power = 0.1; Gain = 14
TRITC (λ = 561 nm) Laser Power = 0.1; Gain = 50
CY5 (λ = 640 nm) Laser Power = 5; Gain = 100

Objective: 4× (3.11 μm/pixel)
FITC (λ = 488 nm) Laser Power = 5; Gain = 25
TRITC (λ = 561 nm) Laser Power = 5; Gain = 50
CY5 (λ = 640 nm) Laser Power = 10; Gain = 100

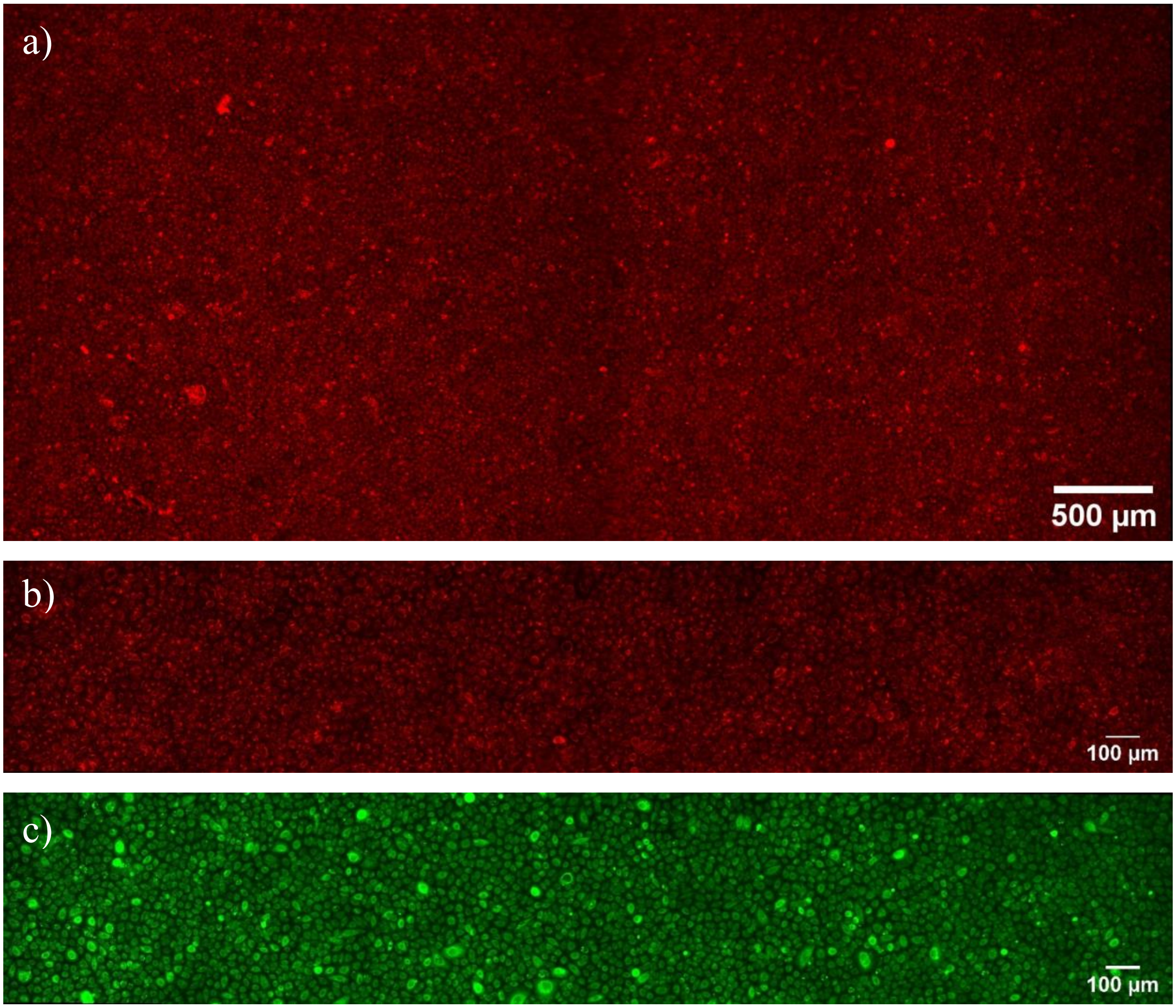

Figure S9. Microscopy images of cells before sliding. a) 4x composite image of cells stained for mucin (red), showing both contacted and non-contacted areas. b) 20x composite image of cells stained for mucin (red) in contact with the probe's sliding path. c) 20x composite image of cells stained with CellTracker™ (green) along the probe's sliding path.

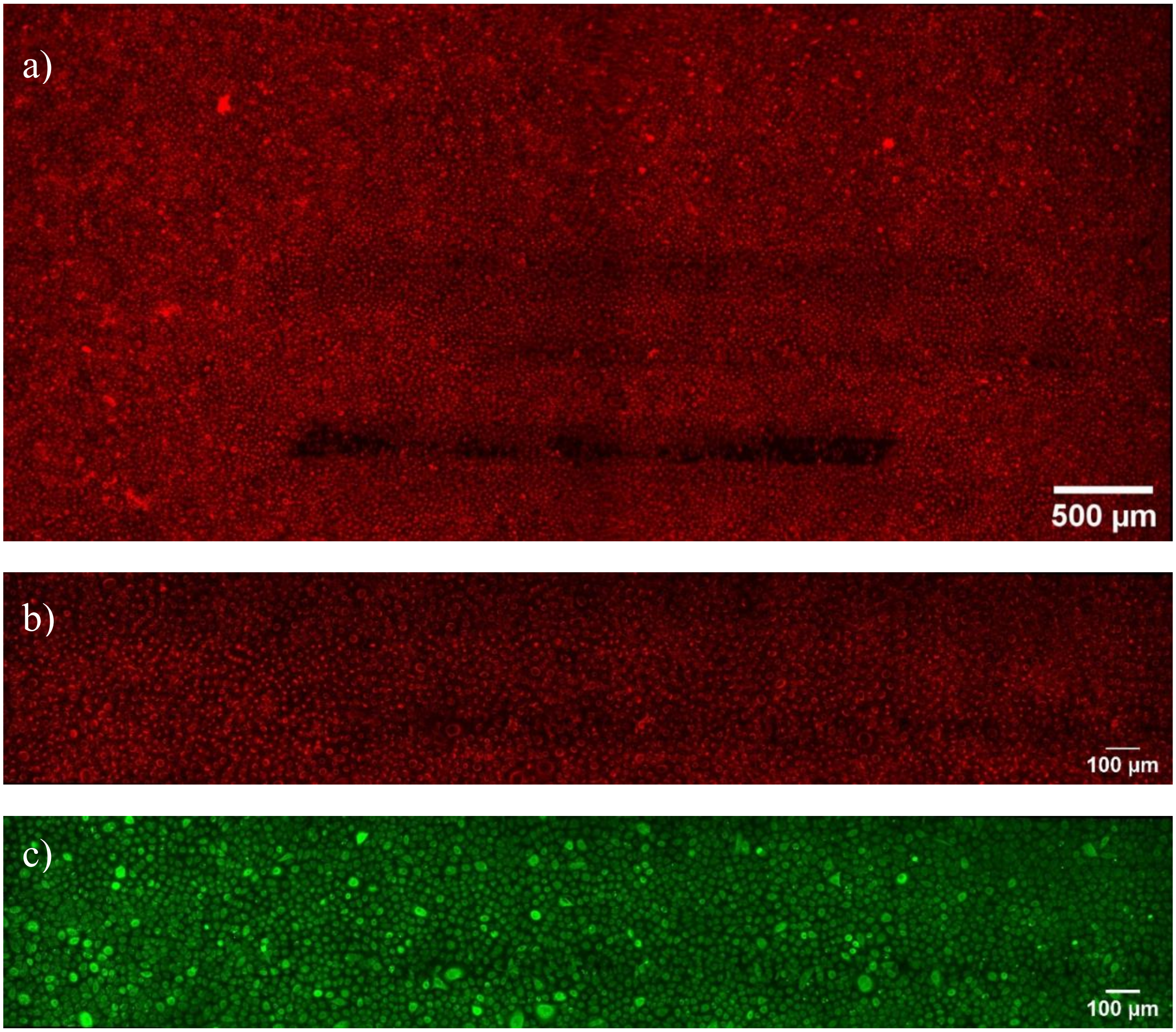

Figure S10 Microscopy images of cells after sliding with a 7.5wt% PAAM gel probe cast against PEEK. a) 4x composite image of cells stained for mucin (red), showing both contacted and non-contacted areas. b) 20x composite image of cells stained for mucin (red) in contact with the probe's sliding path. c) 20x composite image of cells stained with CellTracker™ (green) along the probe's sliding path.

Experiment 5

Probe: PEEK-molded polyacrylamide hydrogel
Countersurface: hTCEpi cell monolayers
Environment: cell growth media, maintained at 37C, 5% CO_2, >95% relative humidity
Normal force: 250 μN
Contact area ~ 0.92 mm^2
Sliding speed: 1 mm/s
Sliding path length (1/2 cycle) = 3 mm
Total sliding distance: 3.6 m
Number of reciprocating cycles: 600
Duration of sliding experiment: 1 h 21 min
Field of view = 620 μm × 3,000 μm

Objective: 20× (0.62 μm/pixel)
FITC (λ = 488 nm) Laser Power = 0.1; Gain = 14
TRITC (λ = 561 nm) Laser Power = 0.1; Gain = 50
CY5 (λ = 640 nm) Laser Power = 5; Gain = 100

Objective: 4× (3.11 μm/pixel)
FITC (λ = 488 nm) Laser Power = 5; Gain = 25
TRITC (λ = 561 nm) Laser Power = 5; Gain = 50
CY5 (λ = 640 nm) Laser Power = 10; Gain = 100

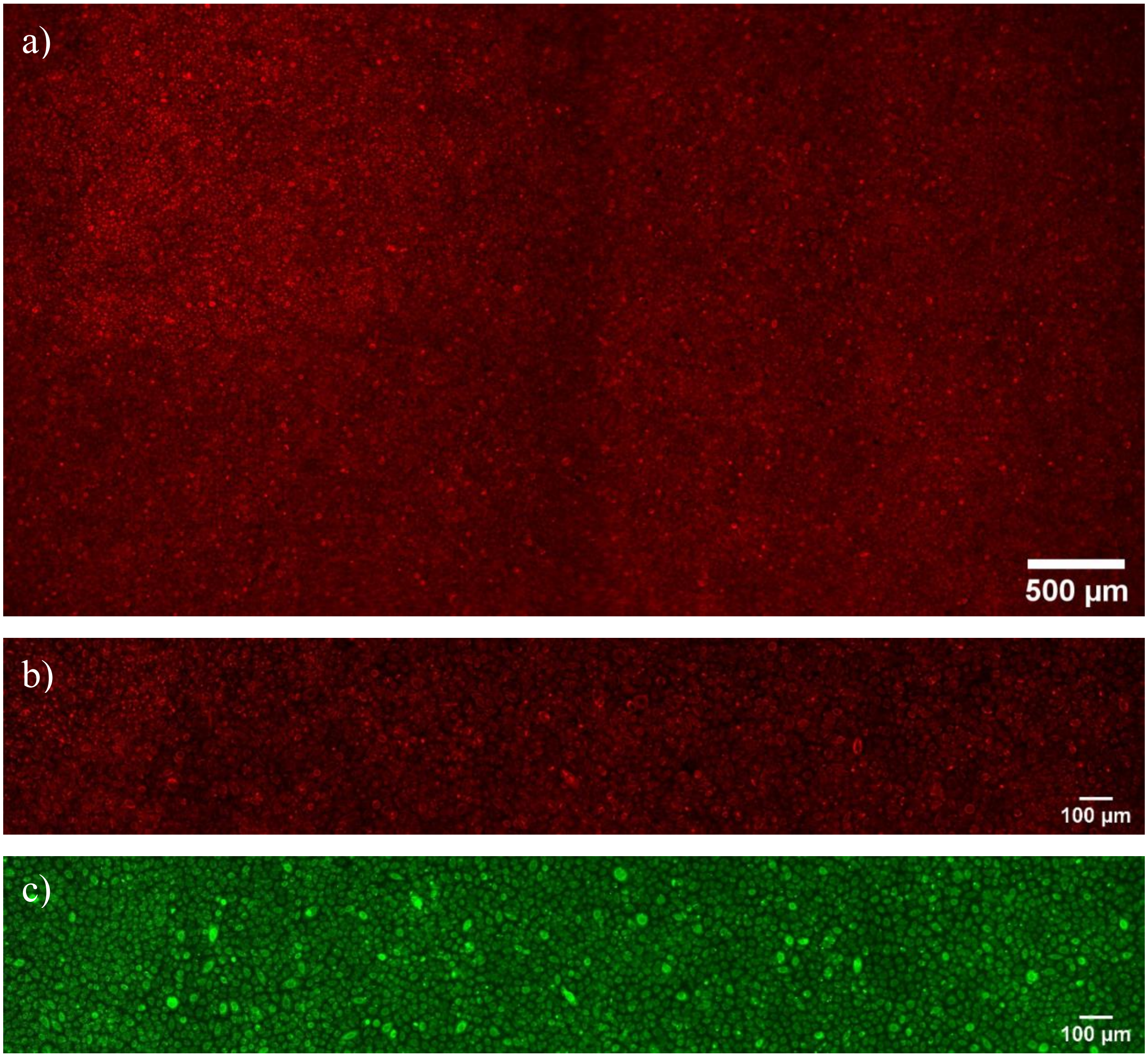

Figure S11. Microscopy images of cells before sliding. a) 4x composite image of cells stained for mucin (red), showing both contacted and non-contacted areas. b) 20x composite image of cells stained for mucin (red) in contact with the probe's sliding path. c) 20x composite image of cells stained with CellTracker™ (green) along the probe's sliding path.

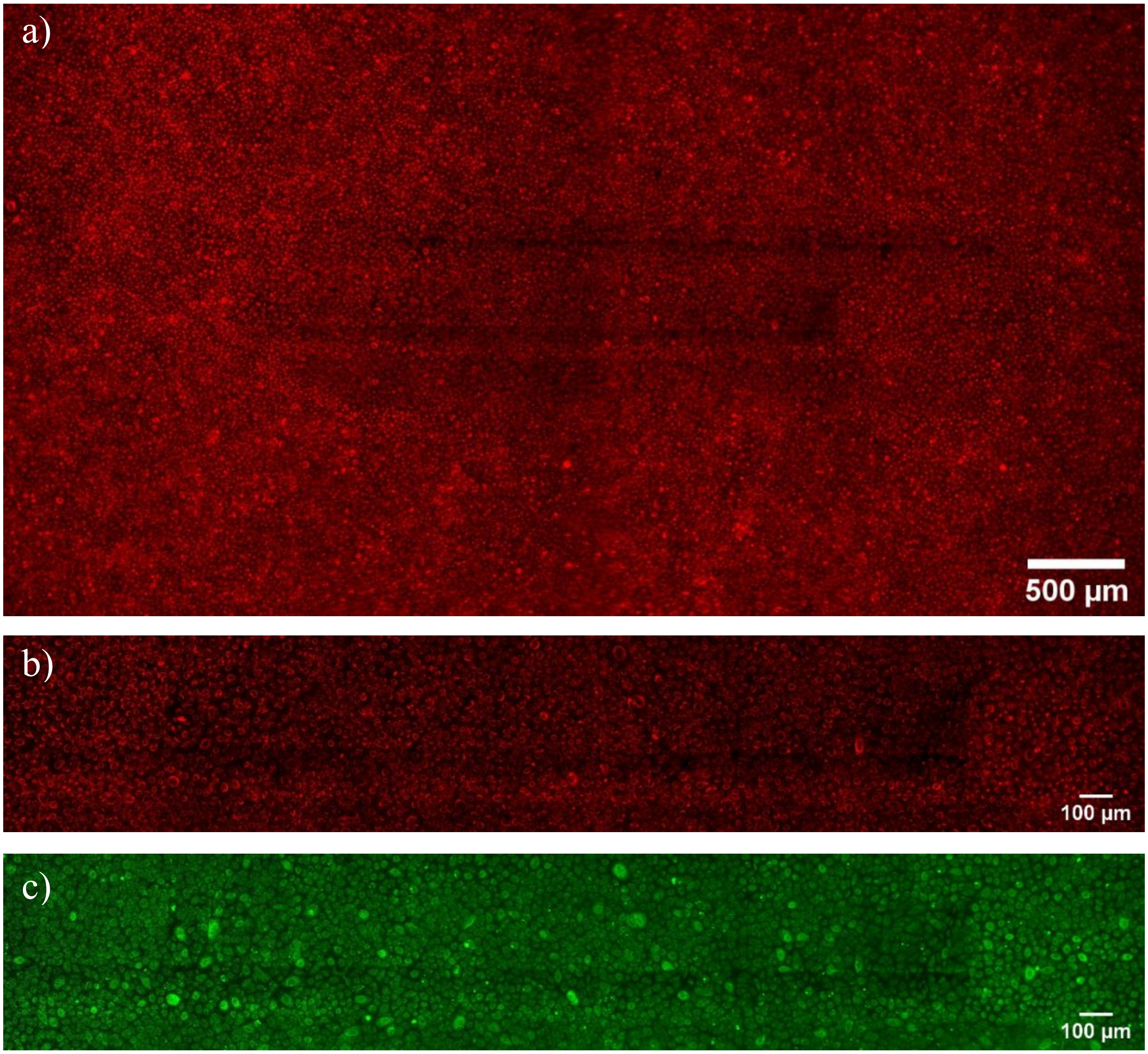

Figure S12. Microscopy images of cells after sliding with a 7.5wt% PAAM gel probe cast against PEEK. a) 4x composite image of cells stained for mucin (red), showing both contacted and non-contacted areas. b) 20x composite image of cells stained for mucin (red) in contact with the probe's sliding path. c) 20x composite image of cells stained with CellTracker™ (green) along the probe's sliding path.

Experiment 6

Probe: PEEK-molded polyacrylamide hydrogel
Countersurface: hTCEpi cell monolayers
Environment: cell growth media, maintained at 37C, 5% CO_2, >95% relative humidity
Normal force: 250 μN
Contact area ~ 0.71 mm^2
Sliding speed: 1 mm/s
Sliding path length (1/2 cycle) = 3 mm
Total sliding distance: 3.6 m
Number of reciprocating cycles: 600
Duration of sliding experiment: 1 h 21 min
Field of view = 620 μm × 3,000 μm

Objective: 20× (0.62 μm/pixel)
FITC (λ = 488 nm) Laser Power = 0.1; Gain = 14
TRITC (λ = 561 nm) Laser Power = 0.1; Gain = 50
CY5 (λ = 640 nm) Laser Power = 4.9; Gain = 100

Objective: 4× (3.11 μm/pixel)
FITC (λ = 488 nm) Laser Power = 5; Gain = 25
TRITC (λ = 561 nm) Laser Power = 5; Gain = 50
CY5 (λ = 640 nm) Laser Power = 10; Gain = 100

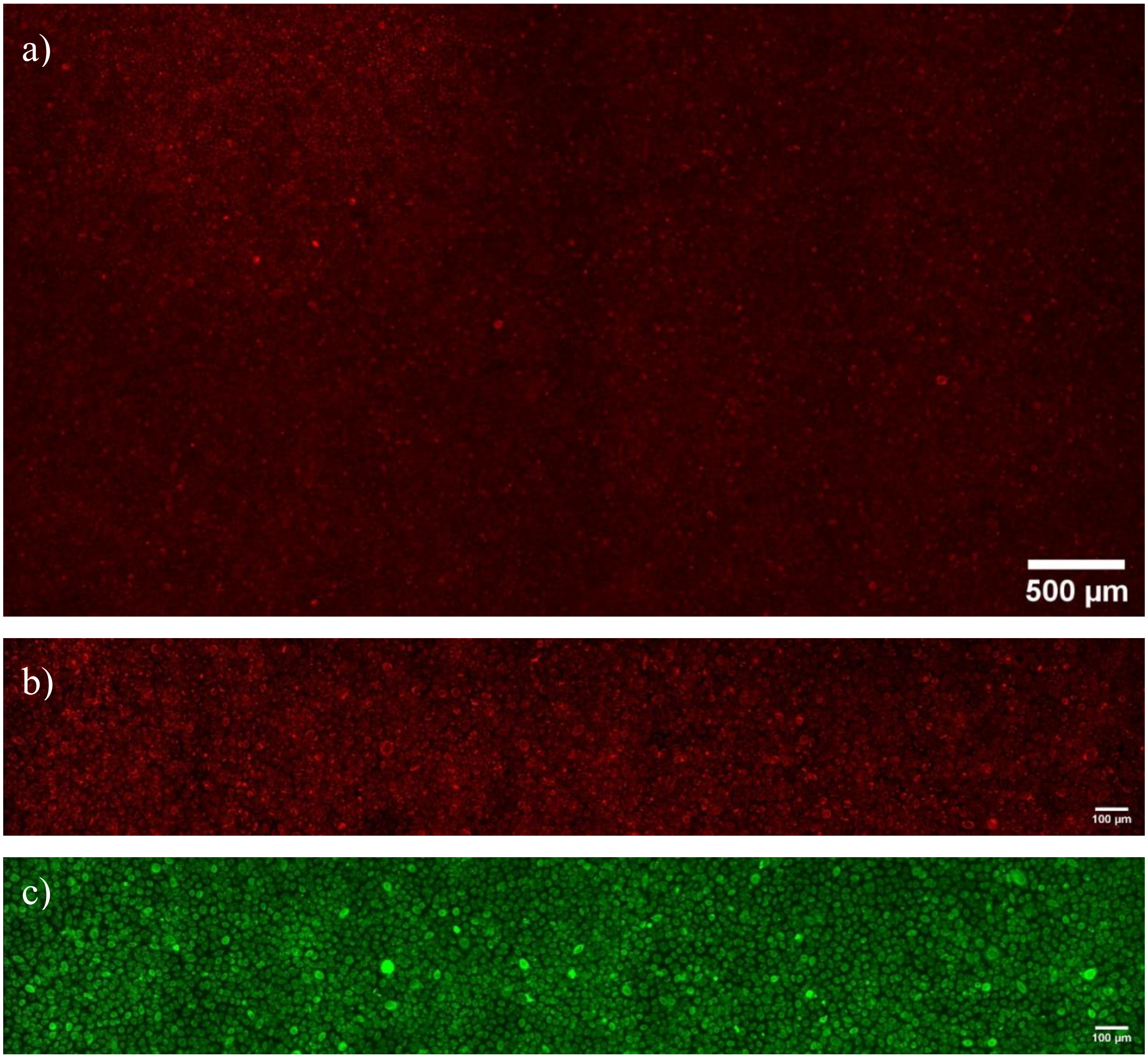

Figure S13. Microscopy images of cells before sliding. a) 4x composite image of cells stained for mucin (red), showing both contacted and non-contacted areas. b) 20x composite image of cells stained for mucin (red) in contact with the probe's sliding path. c) 20x composite image of cells stained with CellTracker™ (green) along the probe's sliding path.

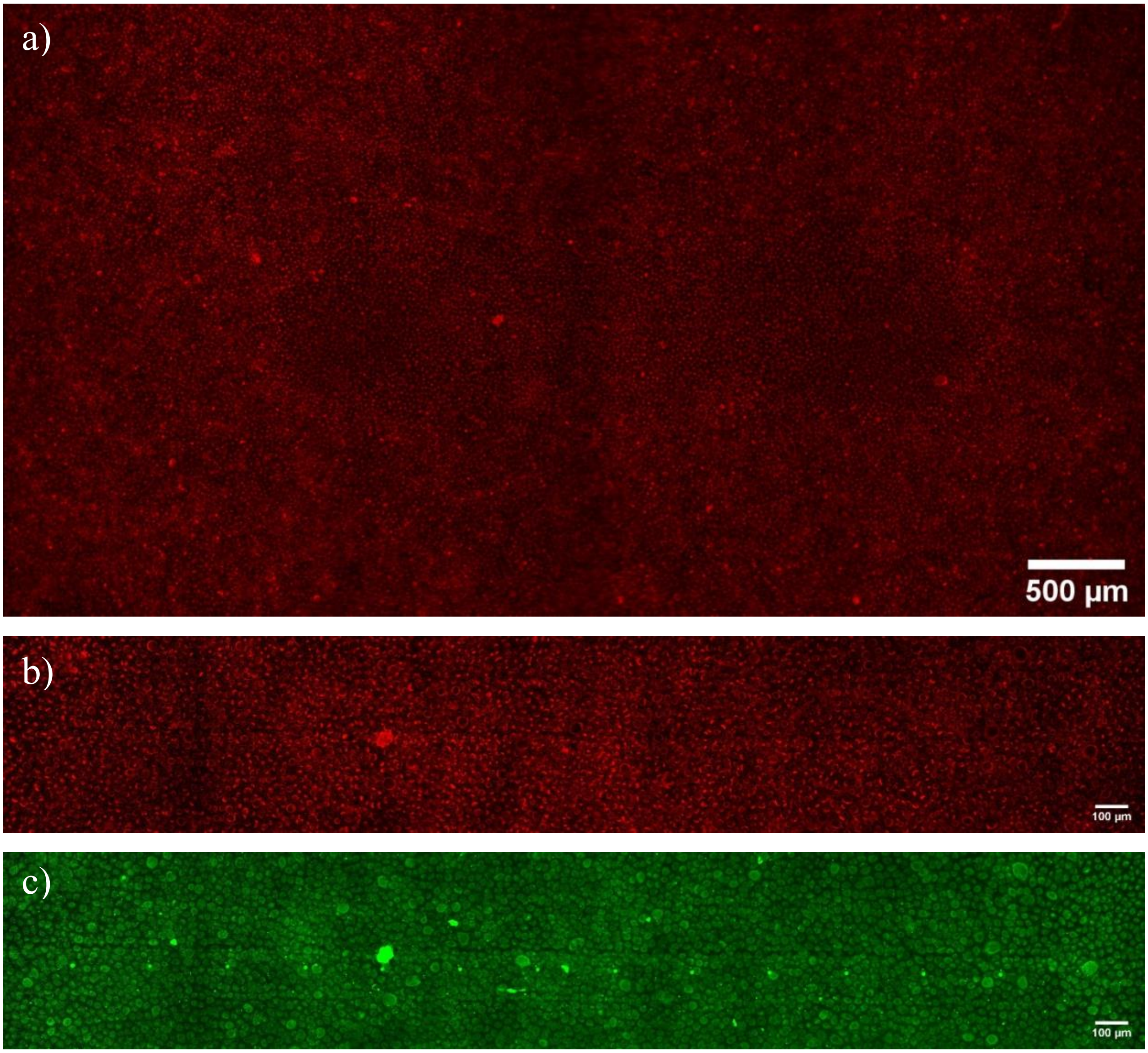

Figure S14 Microscopy images of cells after sliding with a 7.5wt% PAAM gel probe cast against PEEK. a) 4x composite image of cells stained for mucin (red), showing both contacted and non-contacted areas. b) 20x composite image of cells stained for mucin (red) in contact with the probe's sliding path. c) 20x composite image of cells stained with CellTracker™ (green) along the probe's sliding path.

3B. Sliding experiments using PTFE-molded gel probes.

Experiment 1

Probe: PTFE-molded polyacrylamide hydrogel
Countersurface: hTCEpi cell monolayers
Environment: cell growth media, maintained at 37C, 5% CO_2, >95% relative humidity
Normal force: 250 µN
Contact area ~ 1.41 mm^2
Sliding speed: 1 mm/s
Sliding path length (1/2 cycle) = 3 mm
Total sliding distance: 3.6 m
Number of reciprocating cycles: 600
Duration of sliding experiment: 1 h 21 min
Field of view = 620 µm × 3,000 µm

Objective: 20× (0.62 µm/pixel)
FITC (λ = 488 nm) Laser Power = 0.2; Gain = 30
TRITC (λ = 561 nm) Laser Power = 0.12; Gain = 20

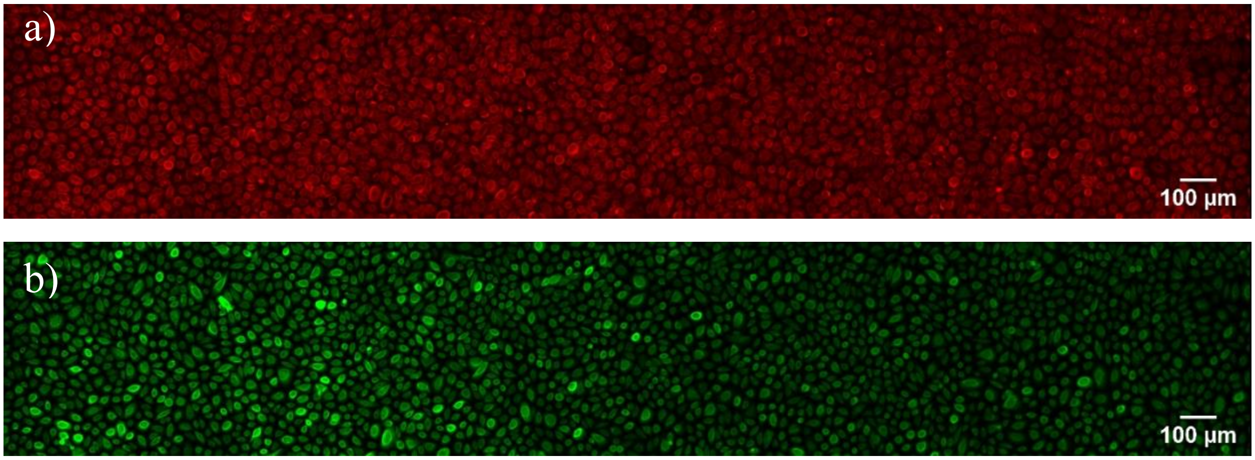

Figure S15. Microscopy images of cells before sliding. a) 20x composite image of cells stained for mucin (red) in contact with the probe's sliding path. b) 20x composite image of cells stained with CellTracker™ (green) along the probe's sliding path

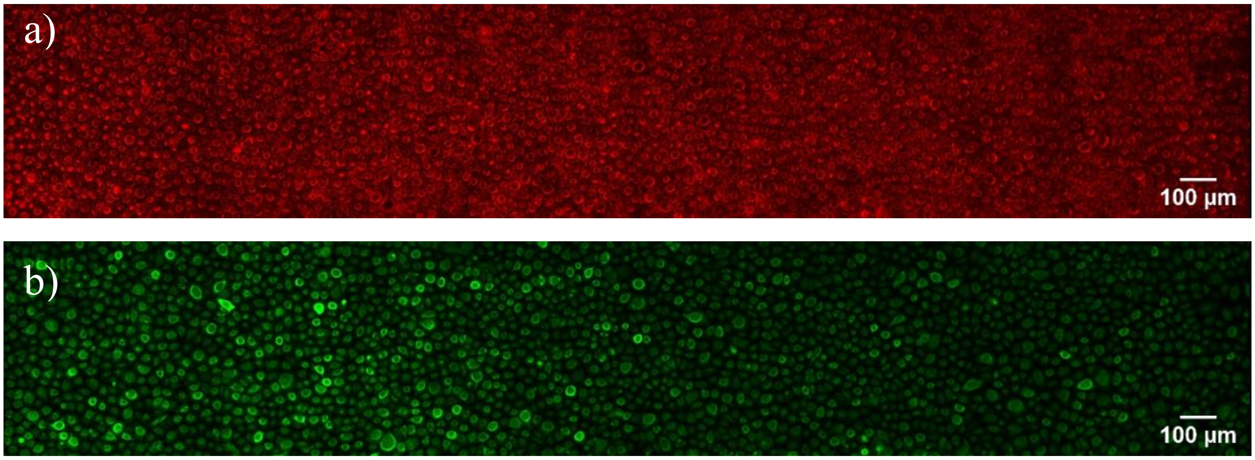

Figure S16. Microscopy images of cells after sliding with a gel probe cast against PTFE. a) 20x composite image of cells stained for mucin (red) in contact with the probe's sliding path. b) 20x composite image of cells stained with CellTracker™ (green) along the probe's sliding path

Experiment 2

Probe: PTFE-molded polyacrylamide hydrogel
Countersurface: hTCEpi cell monolayers
Environment: cell growth media, maintained at 37C, 5% CO_2, >95% relative humidity
Normal force: 250 µN
Contact area ~ 1.41 mm^2
Sliding speed: 1 mm/s
Sliding path length (1/2 cycle) = 3 mm
Total sliding distance: 3.6 m
Number of reciprocating cycles: 600
Duration of sliding experiment: 1 h 21 min
Field of view = 620 µm × 3,000 µm

Objective: 20× (0.62 µm/pixel)
FITC (λ = 488 nm) Laser Power = 0.2; Gain = 35
TRITC (λ = 561 nm) Laser Power = 0.18; Gain = 25

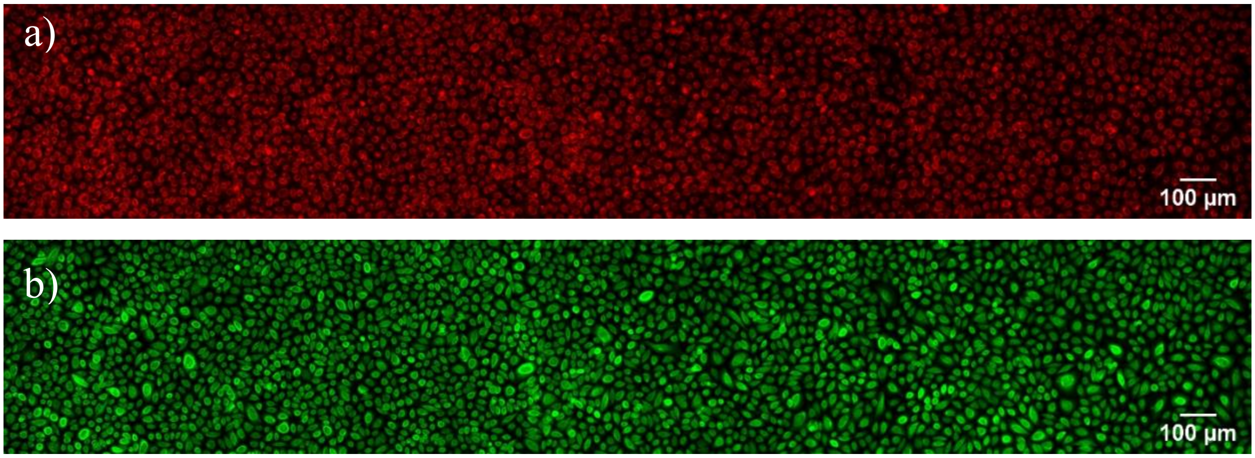

Figure S17. Microscopy images of cells before sliding. a) 20x composite image of cells stained for mucin (red) in contact with the probe's sliding path. b) 20x composite image of cells stained with CellTracker™ (green) along the probe's sliding path

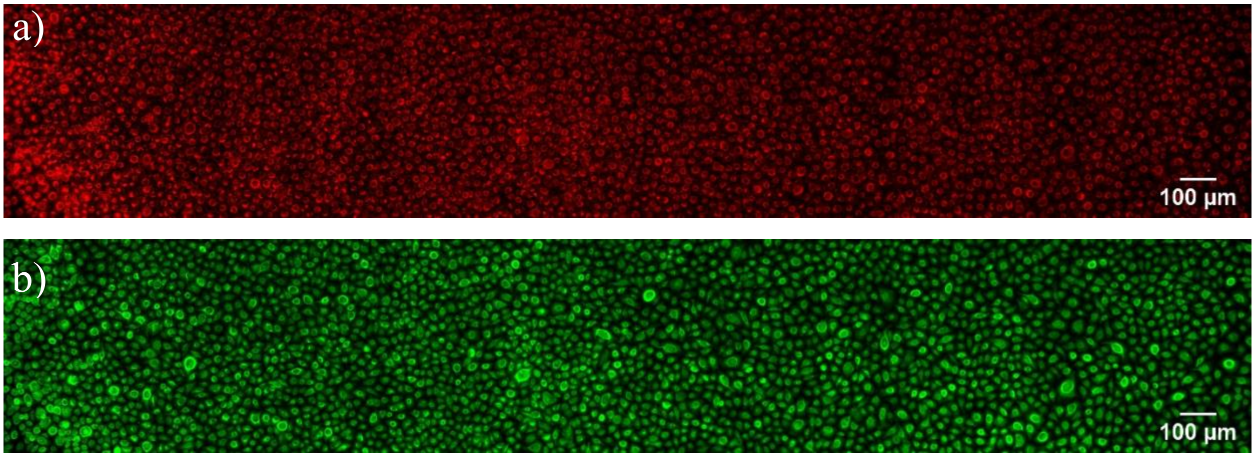

Figure S18. Microscopy images of cells after sliding with a gel probe cast against PTFE. a) 20x composite image of cells stained for mucin (red) in contact with the probe's sliding path. b) 20x composite image of cells stained with CellTracker™ (green) along the probe's sliding path

Experiment 3

Probe: PTFE-molded polyacrylamide hydrogel
Countersurface: hTCEpi cell monolayers
Environment: cell growth media, maintained at 37C, 5% CO_2, >95% relative humidity
Normal force: 250 µN
Contact area ~ 1.41 mm^2
Sliding speed: 1 mm/s
Sliding path length (1/2 cycle) = 3 mm
Total sliding distance: 3.6 m
Number of reciprocating cycles: 600
Duration of sliding experiment: 1 h 21 min
Field of view = 620 µm × 3,000 µm

Objective: 20× (0.62 µm/pixel)
FITC (λ = 488 nm) Laser Power = 0.15; Gain = 25
TRITC (λ = 561 nm) Laser Power = 0.15; Gain = 25

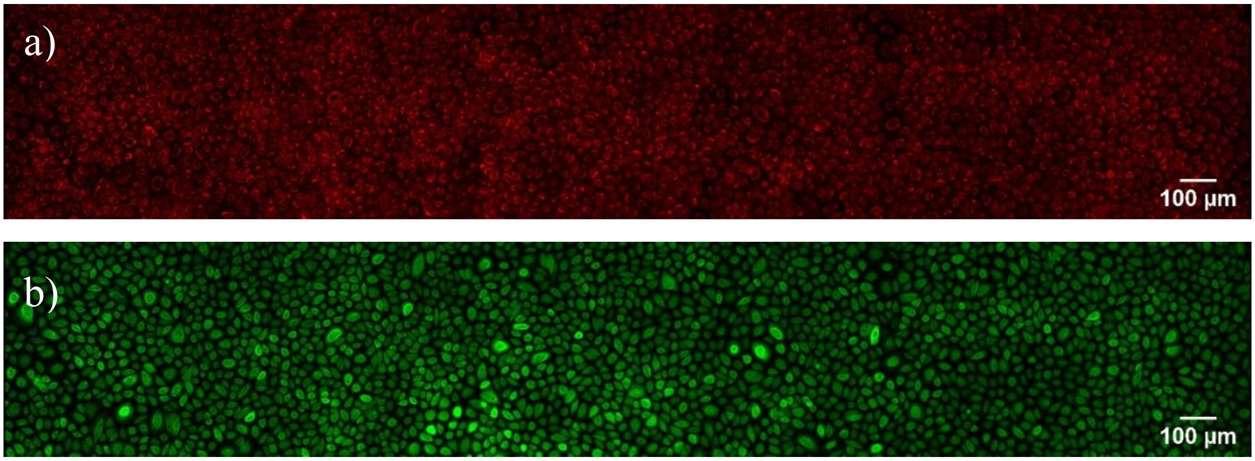

Figure S19. Microscopy images of cells before sliding. a) 20x composite image of cells stained for mucin (red) in contact with the probe's sliding path. b) 20x composite image of cells stained with CellTracker™ (green) along the probe's sliding path

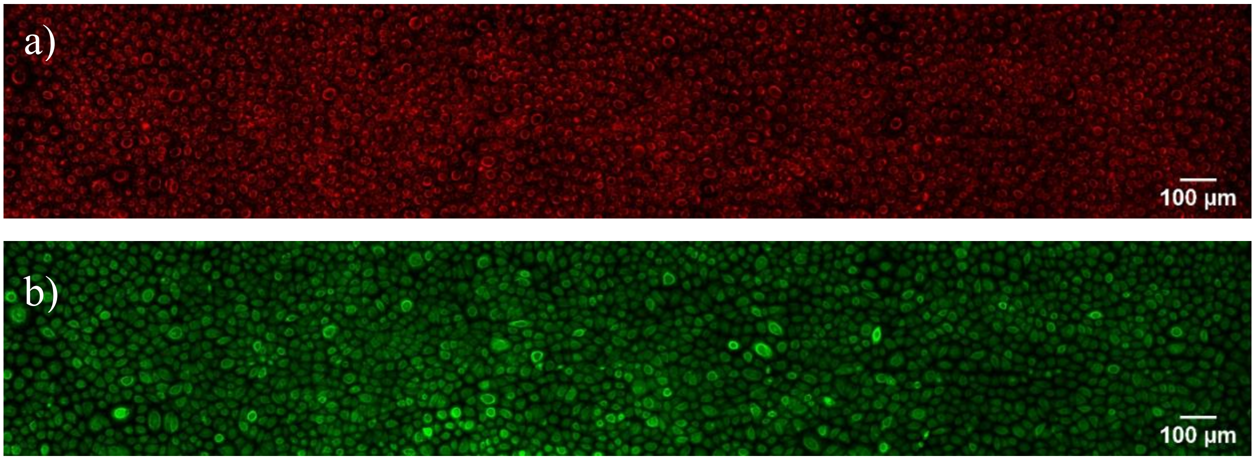

Figure S20. Microscopy images of cells after sliding with a gel probe cast against PTFE. a) 20x composite image of cells stained for mucin (red) in contact with the probe's sliding path. b) 20x composite image of cells stained with CellTracker™ (green) along the probe's sliding path

Experiment 4

Probe: PTFE-molded polyacrylamide hydrogel
Countersurface: hTCEpi cell monolayers
Environment: cell growth media, maintained at 37C, 5% CO_2, >95% relative humidity
Normal force: 250 μN
Contact area ~ 1.24 mm^2
Sliding speed: 1 mm/s
Sliding path length (1/2 cycle) = 3 mm
Total sliding distance: 3.6 m
Number of reciprocating cycles: 600
Duration of sliding experiment: 1 h 21 min
Field of view = 620 μm × 3,000 μm

Objective: 20× (0.62 μm/pixel)
FITC (λ = 488 nm) Laser Power = 0.1; Gain = 14
TRITC (λ = 561 nm) Laser Power = 0.1; Gain = 25
CY5 (λ = 640 nm) Laser Power = 3; Gain = 85

Objective: 4× (3.11 μm/pixel)
FITC (λ = 488 nm) Laser Power = 5; Gain = 25
TRITC (λ = 561 nm) Laser Power = 5; Gain = 50
CY5 (λ = 640 nm) Laser Power = 10; Gain = 100

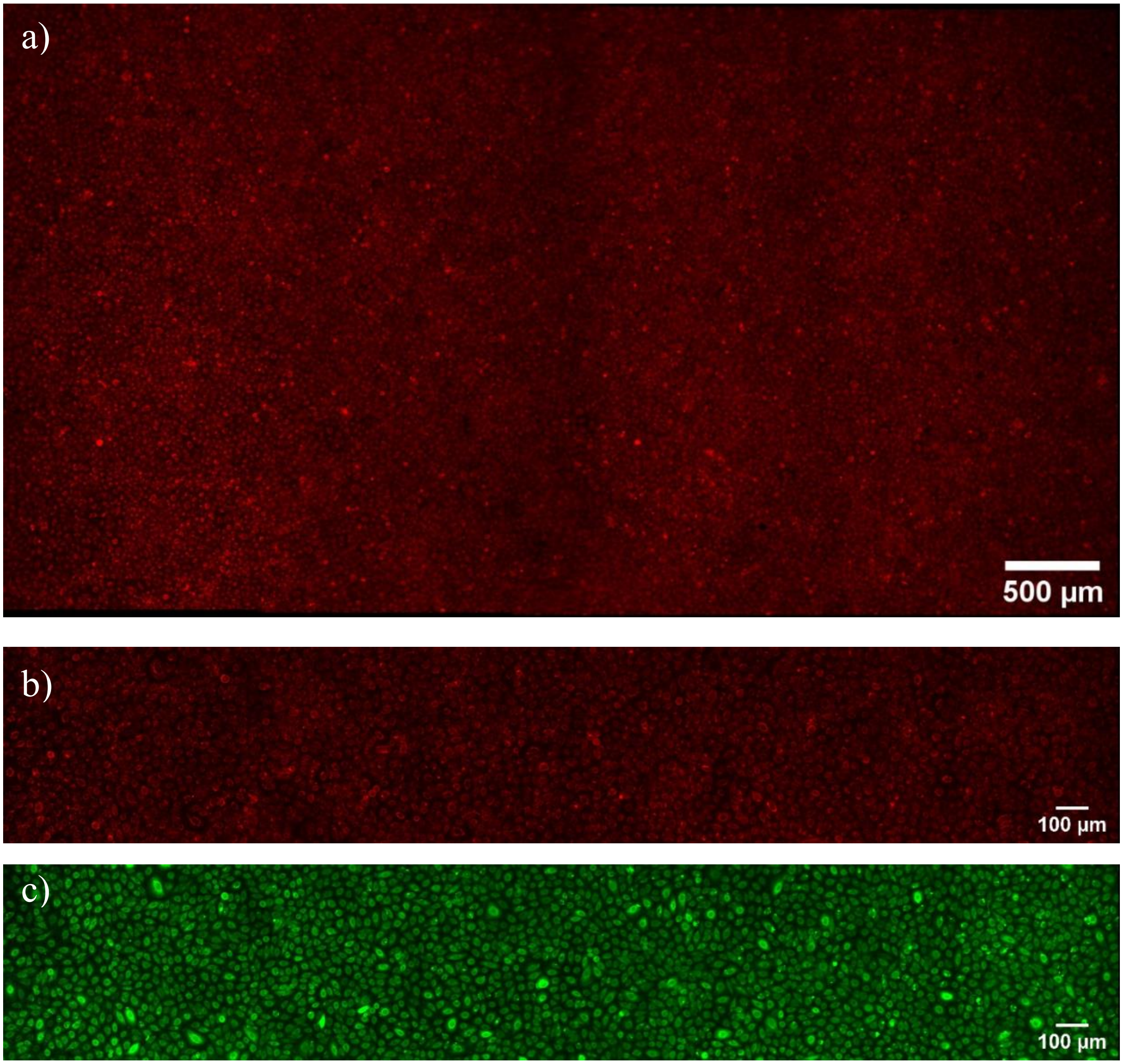

Figure S21. Microscopy images of cells before sliding. a) 4x composite image of cells stained for mucin (red), showing both contacted and non-contacted areas. b) 20x composite image of cells stained for mucin (red) in contact with the probe's sliding path. c) 20x composite image of cells stained with CellTracker™ (green) along the probe's sliding path.

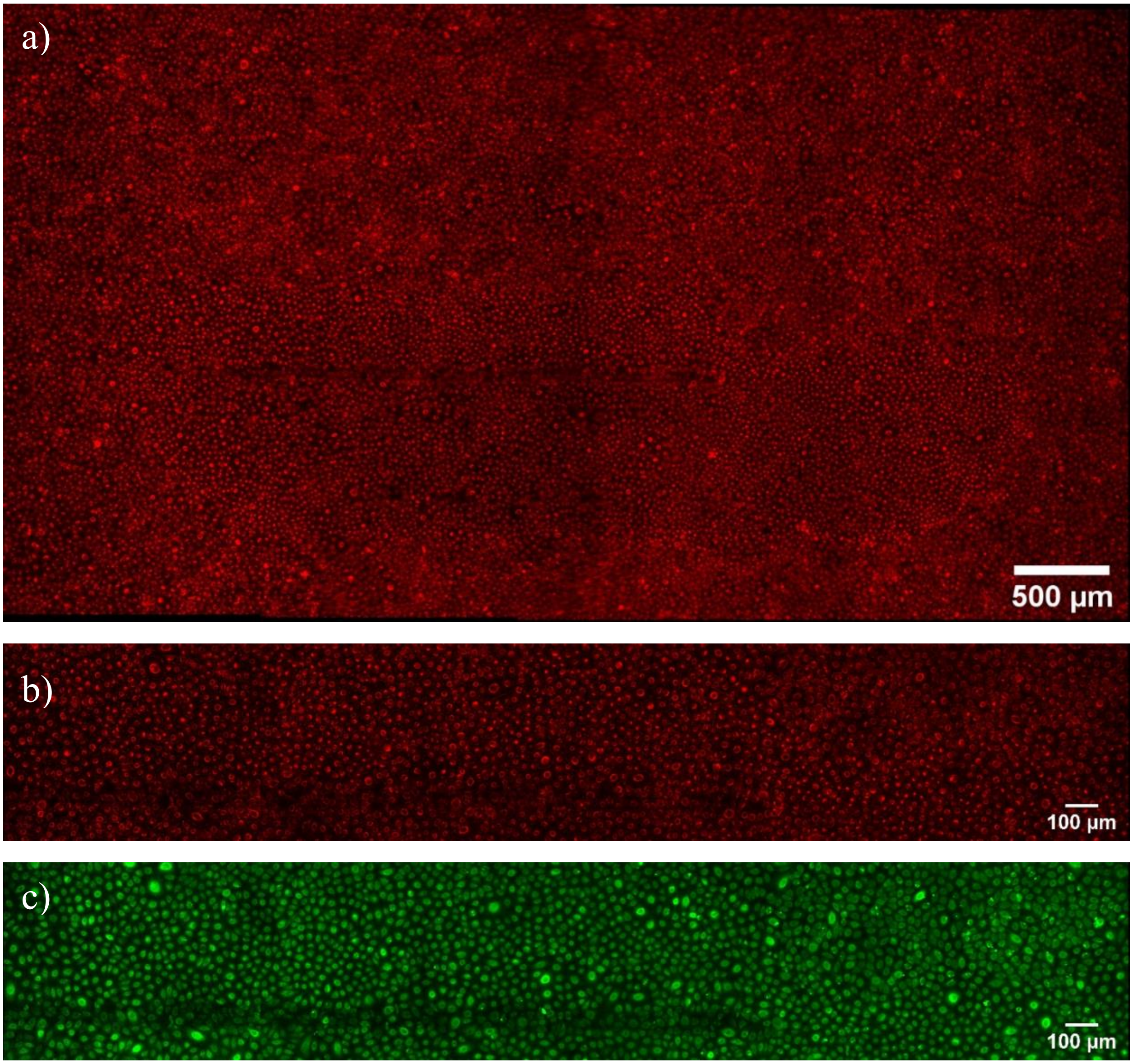

Figure S22. Microscopy images of cells after sliding with a 7.5wt% PAAM gel probe cast against PTFE. a) 4x composite image of cells stained for mucin (red), showing both contacted and non-contacted areas. b) 20x composite image of cells stained for mucin (red) in contact with the probe's sliding path. c) 20x composite image of cells stained with CellTracker™ (green) along the probe's sliding path.

Experiment 5

Probe: PTFE-molded polyacrylamide hydrogel
Countersurface: hTCEpi cell monolayers
Environment: cell growth media, maintained at 37C, 5% CO_2, >95% relative humidity
Normal force: 250 μN
Contact area ~ 1.33 mm^2
Sliding speed: 1 mm/s
Sliding path length (1/2 cycle) = 3 mm
Total sliding distance: 3.6 m
Number of reciprocating cycles: 600
Duration of sliding experiment: 1 h 21 min
Field of view = 620 μm × 3,000 μm

Objective: 20× (0.62 μm/pixel)
FITC (λ = 488 nm) Laser Power = 0.1; Gain = 14
TRITC (λ = 561 nm) Laser Power = 0.1; Gain = 20
CY5 (λ = 640 nm) Laser Power = 3; Gain = 85

Objective: 4× (3.11 μm/pixel)
FITC (λ = 488 nm) Laser Power = 5; Gain = 25
TRITC (λ = 561 nm) Laser Power = 5; Gain = 50
CY5 (λ = 640 nm) Laser Power = 10; Gain = 100

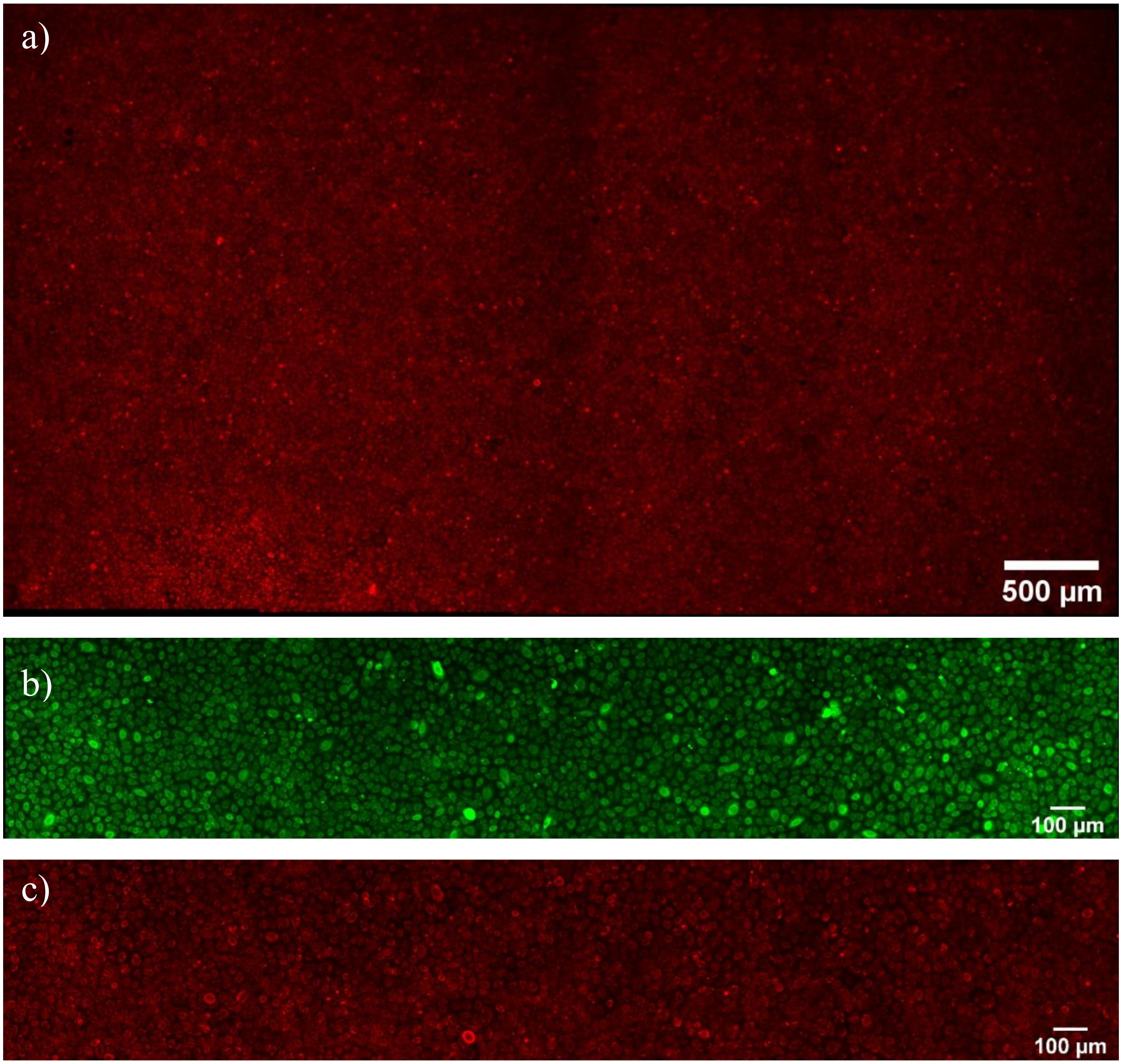

Figure S23. Microscopy images of cells before sliding. a) 4x composite image of cells stained for mucin (red), showing both contacted and non-contacted areas. b) 20x composite image of cells stained for mucin (red) in contact with the probe's sliding path. c) 20x composite image of cells stained with CellTracker™ (green) along the probe's sliding path.

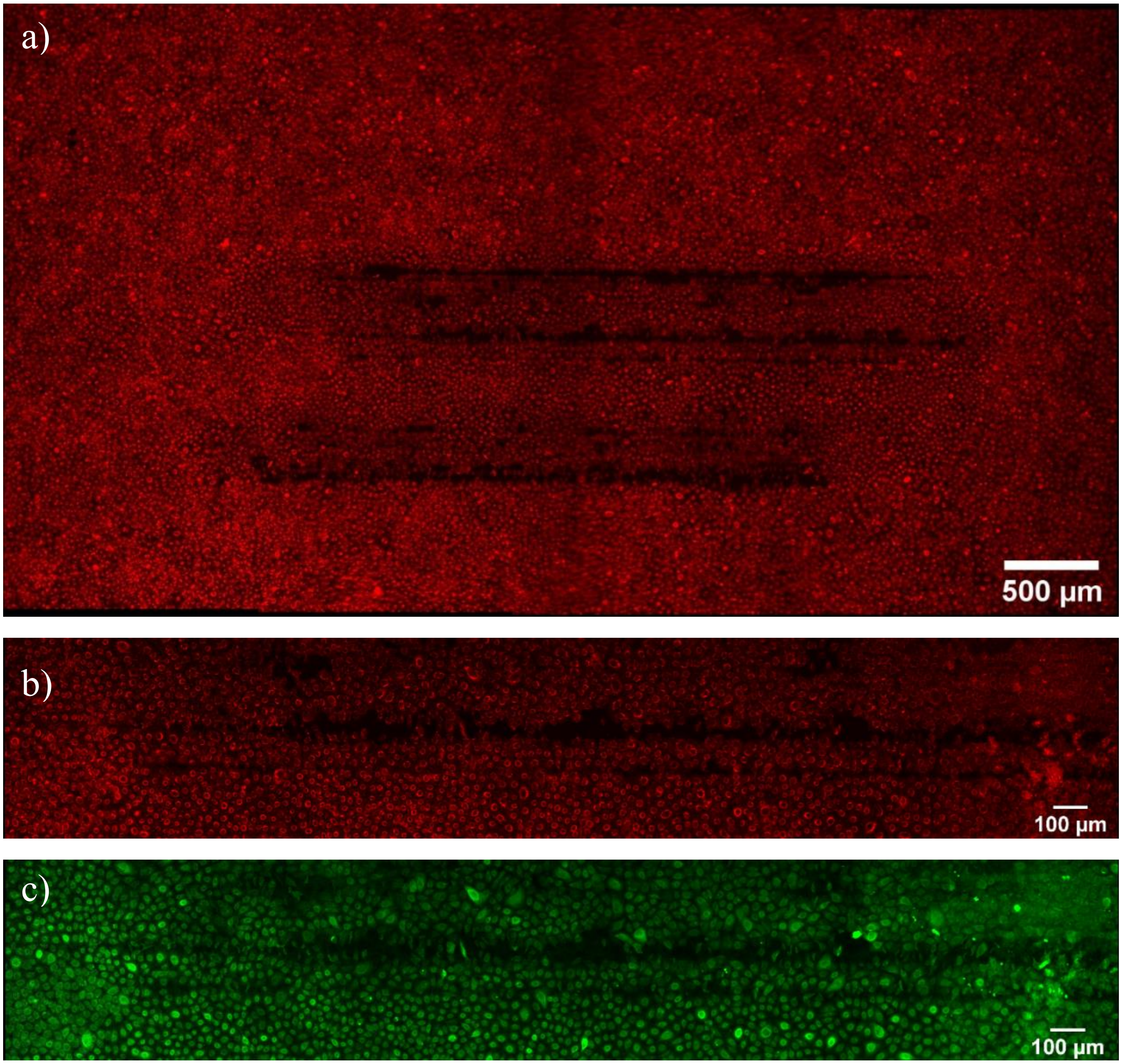

Figure S24. Microscopy images of cells after sliding with a 7.5wt% PAAM gel probe cast against PTFE. a) 4x composite image of cells stained for mucin (red), showing both contacted and non-contacted areas. b) 20x composite image of cells stained for mucin (red) in contact with the probe's sliding path. c) 20x composite image of cells stained with CellTracker™ (green) along the probe's sliding path.

Experiment 6

Probe: PTFE-molded polyacrylamide hydrogel
Countersurface: hTCEpi cell monolayers
Environment: cell growth media, maintained at 37C, 5% CO_2, >95% relative humidity
Normal force: 250 μN
Contact area ~ 0.71 mm^2
Sliding speed: 1 mm/s
Sliding path length (1/2 cycle) = 3 mm
Total sliding distance: 3.6 m
Number of reciprocating cycles: 600
Duration of sliding experiment: 1 h 21 min
Field of view = 620 μm × 3,000 μm

Objective: 20× (0.62 μm/pixel)
FITC (λ = 488 nm) Laser Power = 0.1; Gain = 14
TRITC (λ = 561 nm) Laser Power = 0.1; Gain = 50
CY5 (λ = 640 nm) Laser Power = 5; Gain = 100

Objective: 4× (3.11 μm/pixel)
FITC (λ = 488 nm) Laser Power = 5; Gain = 25
TRITC (λ = 561 nm) Laser Power = 5; Gain = 50
CY5 (λ = 640 nm) Laser Power = 10; Gain = 100

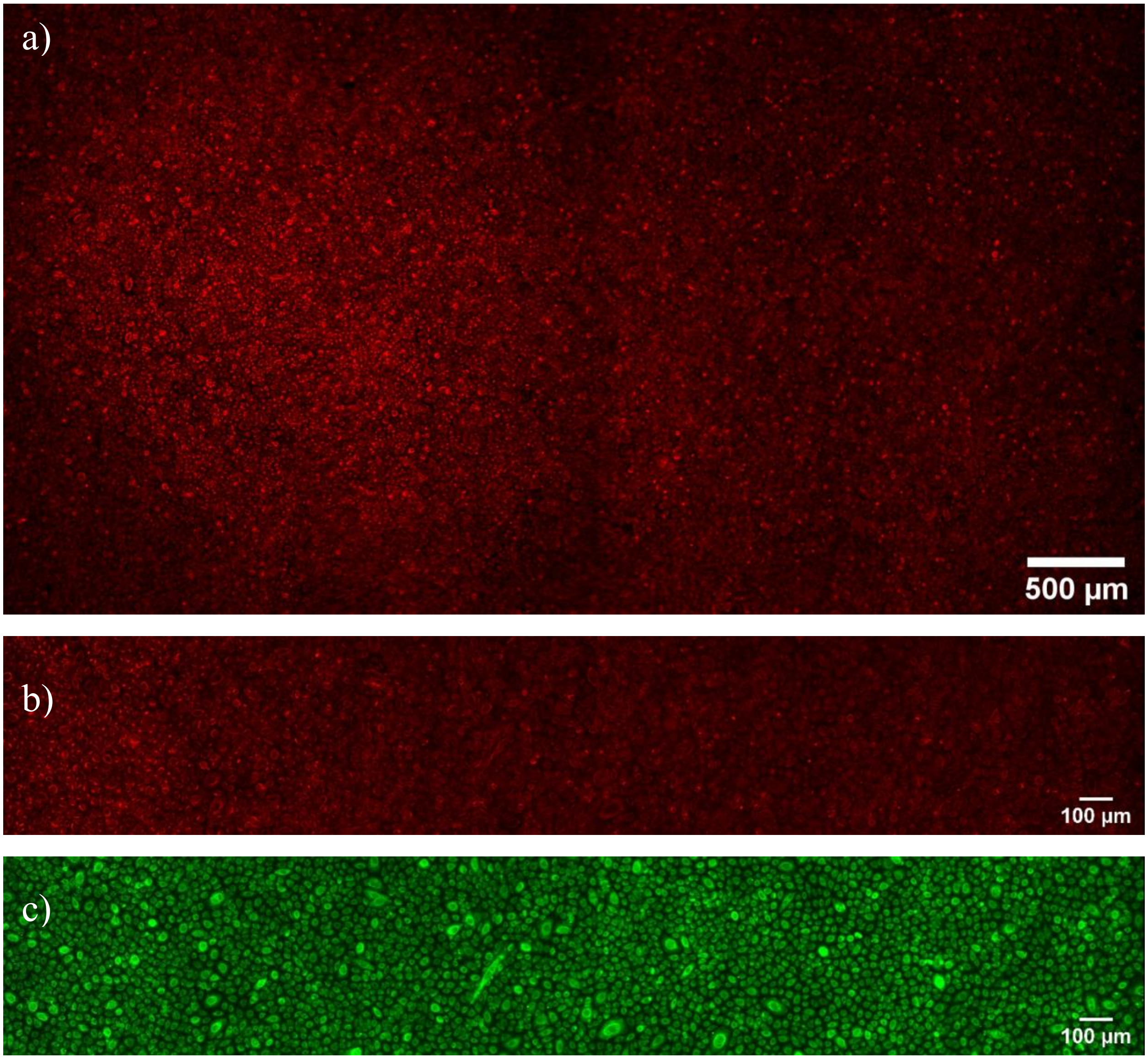

Figure S25. Microscopy images of cells before sliding. a) 4x composite image of cells stained for mucin (red), showing both contacted and non-contacted areas. b) 20x composite image of cells stained for mucin (red) in contact with the probe's sliding path. c) 20x composite image of cells stained with CellTracker™ (green) along the probe's sliding path.

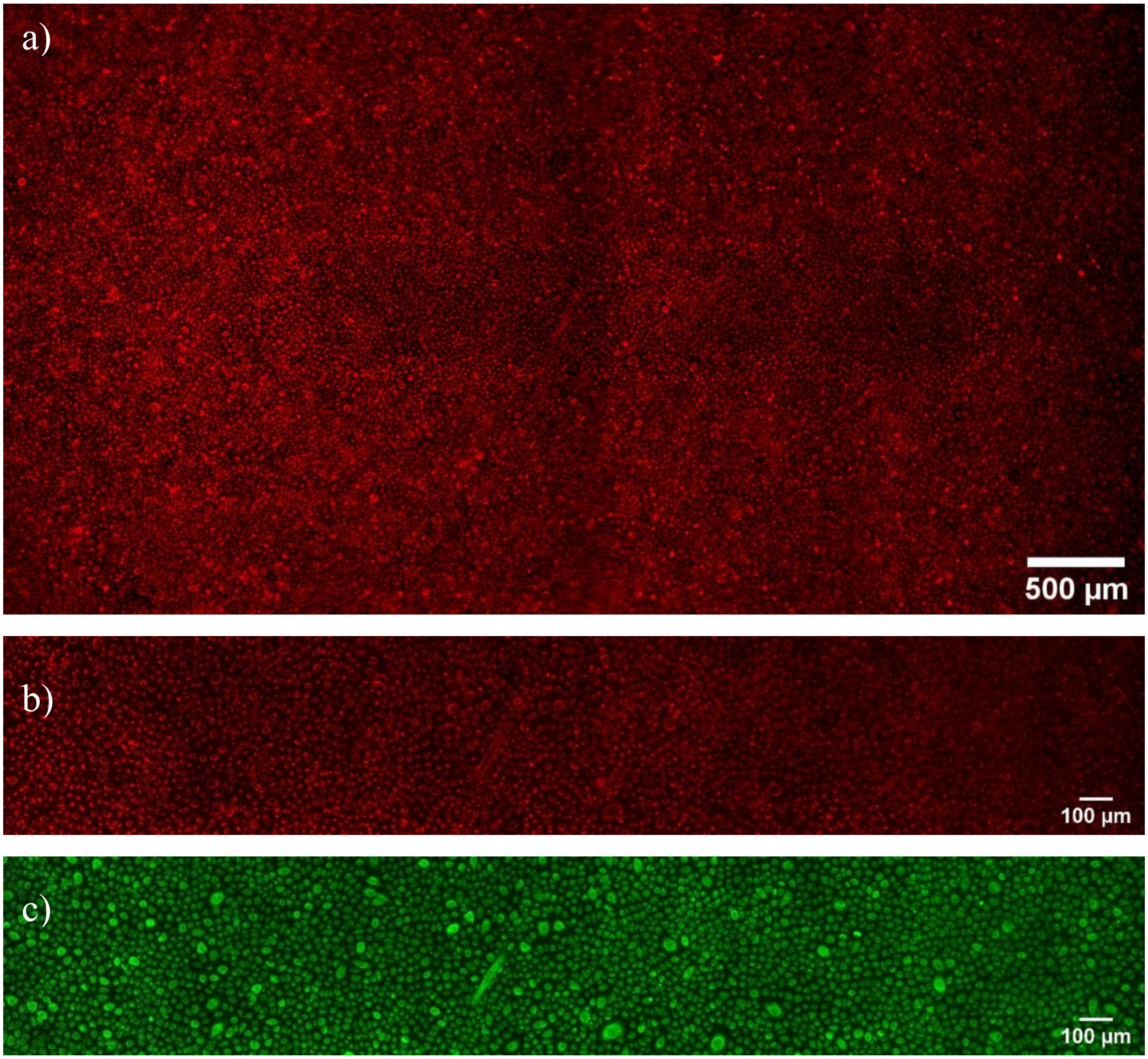

Figure S26. Microscopy images of cells after sliding with a 7.5wt% PAAM gel probe cast against PTFE. a) 4x composite image of cells stained for mucin (red), showing both contacted and non-contacted areas. b) 20x composite image of cells stained for mucin (red) in contact with the probe's sliding path. c) 20x composite image of cells stained with CellTracker™ (green) along the probe's sliding path.

3C. Sliding experiments using glass-molded gel probes.

Experiment 1

Probe: glass-molded polyacrylamide hydrogel
Countersurface: hTCEpi cell monolayers
Environment: cell growth media, maintained at 37C, 5% CO_2, >95% relative humidity
Normal force: 250 µN
Contact area ~ 0.32 mm^2
Sliding speed: 1 mm/s
Sliding path length (1/2 cycle) = 3 mm
Total sliding distance: 3.6 m
Number of reciprocating cycles: 600
Duration of sliding experiment: 1 h 21 min
Field of view = 620 µm × 3,000 µm

Objective: 20× (0.62 µm/pixel)
FITC (λ = 488 nm) Laser Power = 0.1; Gain = 15
CY5 (λ = 640 nm) Laser Power = 3.0; Gain = 70

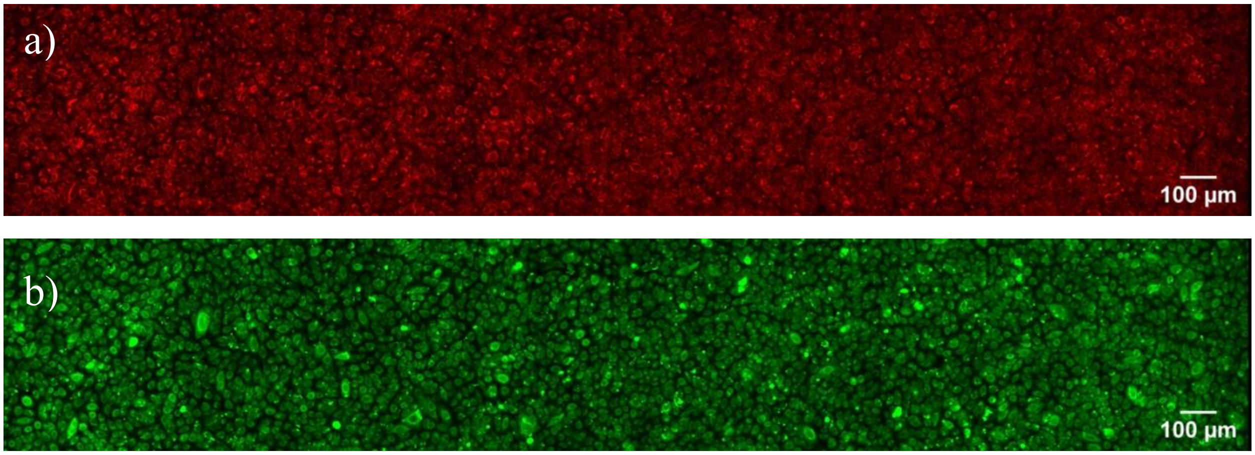

Figure S27. Microscopy images of cells before sliding. a) 20x composite image of cells stained for mucin (red) in contact with the probe's sliding path. b) 20x composite image of cells stained with CellTracker™ (green) along the probe's sliding path

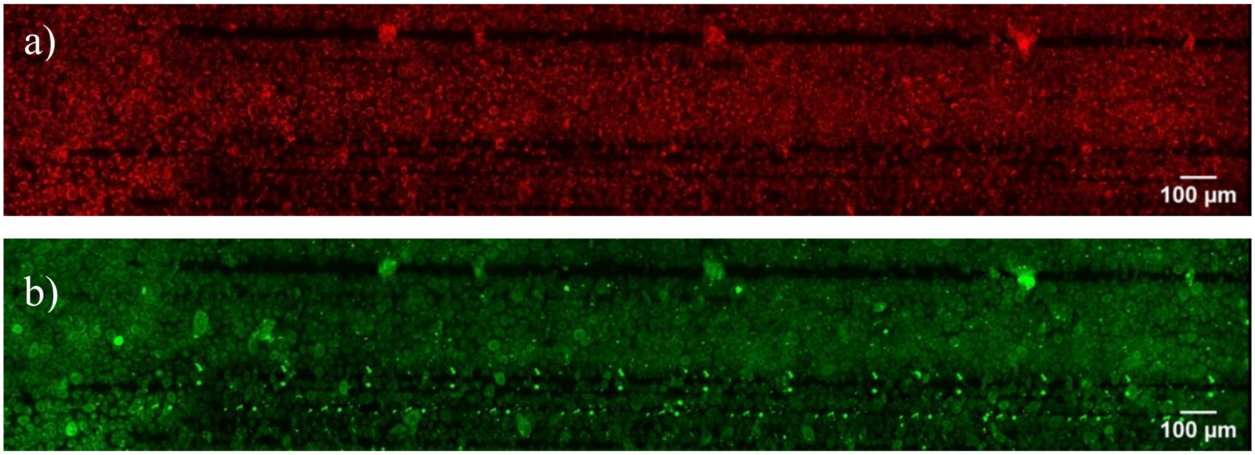

Figure S28. Microscopy images of cells after sliding with a 7.5 wt% PAAM gel probe cast against glass. a) 20x composite image of cells stained for mucin (red) in contact with the probe's sliding path. b) 20x composite image of cells stained with CellTracker™ (green) along the probe's sliding path

Experiment 2

Probe: glass-molded polyacrylamide hydrogel
Countersurface: hTCEpi cell monolayers
Environment: cell growth media, maintained at 37C, 5% CO_2, >95% relative humidity
Normal force: 250 μN
Contact area ~ 0.32 mm^2
Sliding speed: 1 mm/s
Sliding path length (1/2 cycle) = 3 mm
Total sliding distance: 3.6 m
Number of reciprocating cycles: 600
Duration of sliding experiment: 1 h 21 min
Field of view = 620 μm × 3,000 μm

Objective: 20× (0.62 μm/pixel)
FITC (λ = 488 nm) Laser Power = 0.1; Gain = 20
CY5 (λ = 640 nm) Laser Power = 3.0; Gain = 65

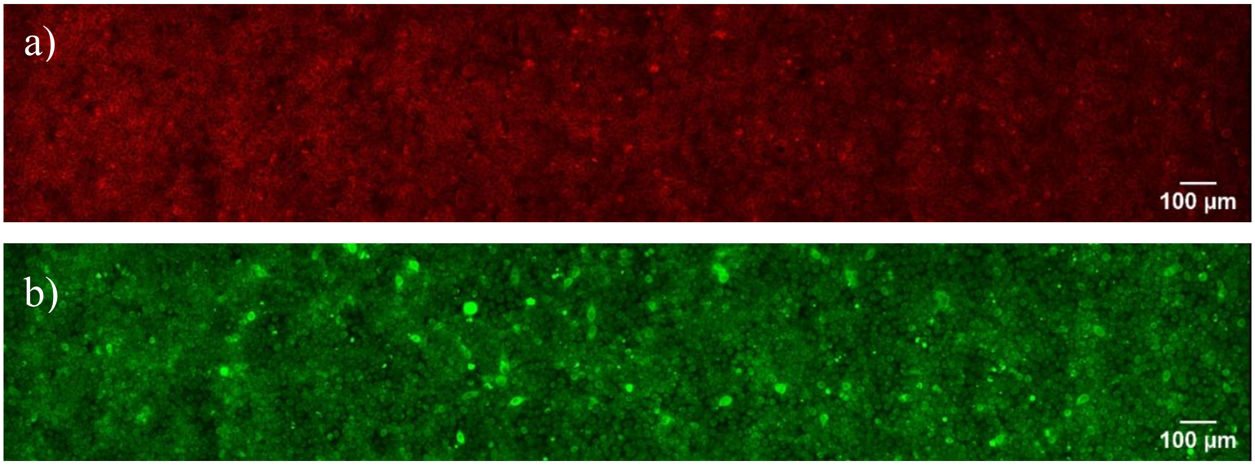

Figure S29. Microscopy images of cells before sliding. a) 20x composite image of cells stained for mucin (red) in contact with the probe's sliding path. b) 20x composite image of cells stained with CellTracker™ (green) along the probe's sliding path

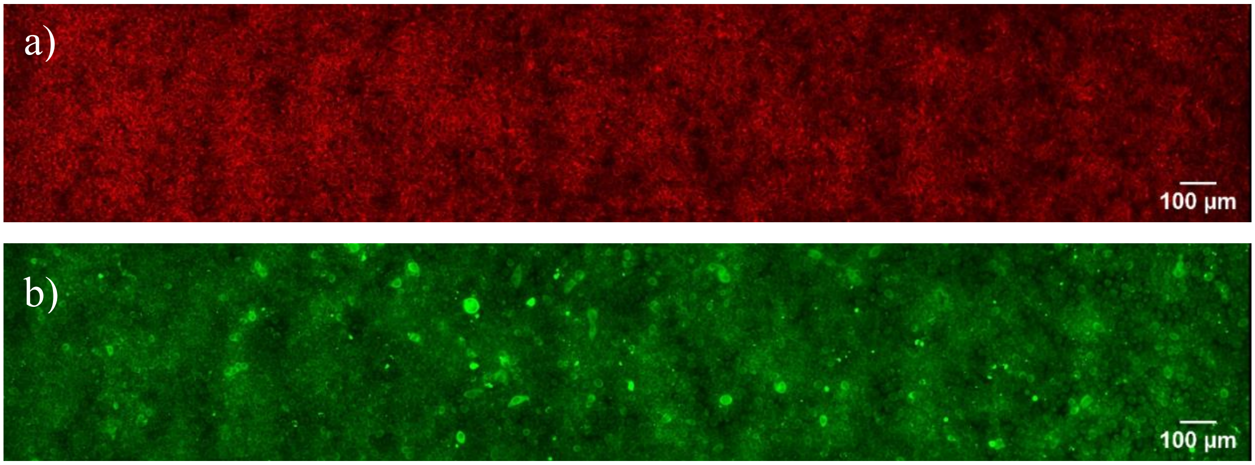

Figure S30. Microscopy images of cells after sliding with a 7.5 wt% PAAM gel probe cast against glass. a) 20x composite image of cells stained for mucin (red) in contact with the probe's sliding path. b) 20x composite image of cells stained with CellTracker™ (green) along the probe's sliding path

Experiment 3

Probe: glass-molded polyacrylamide hydrogel
Countersurface: hTCEpi cell monolayers
Environment: cell growth media, maintained at 37C, 5% CO_2, >95% relative humidity
Normal force: 250 µN
Contact area ~ 0.32 mm^2
Sliding speed: 1 mm/s
Sliding path length (1/2 cycle) = 3 mm
Total sliding distance: 3.6 m
Number of reciprocating cycles: 600
Duration of sliding experiment: 1 h 21 min
Field of view = 620 µm × 3,000 µm

Objective: 20× (0.62 µm/pixel)
FITC (λ = 488 nm) Laser Power = 0.1; Gain = 20
CY5 (λ = 640 nm) Laser Power = 3.0; Gain = 65

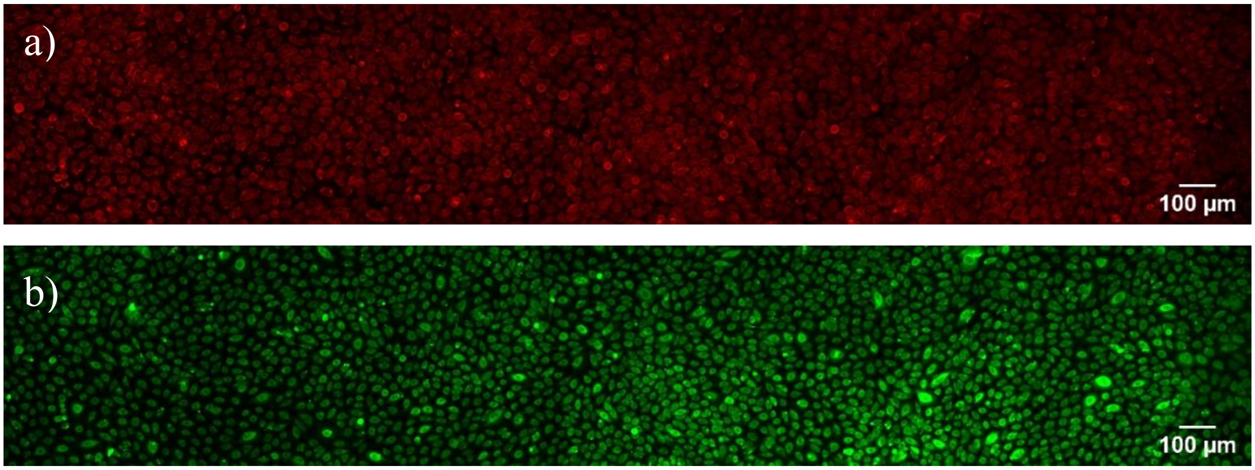

Figure S31 Microscopy images of cells before sliding. a) 20x composite image of cells stained for mucin (red) in contact with the probe's sliding path. b) 20x composite image of cells stained with CellTracker™ (green) along the probe's sliding path

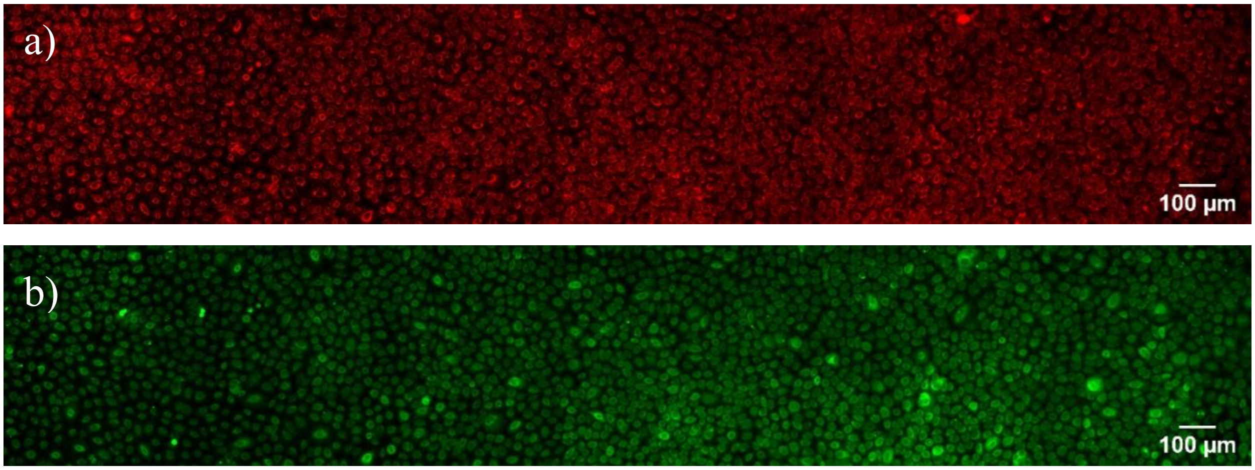

Figure S32. Microscopy images of cells after sliding with a 7.5 wt% PAAM gel probe cast against glass. a) 20x composite image of cells stained for mucin (red) in contact with the probe's sliding path. b) 20x composite image of cells stained with CellTracker™ (green) along the probe's sliding path

Experiment 4

Probe: glass-molded polyacrylamide hydrogel
Countersurface: hTCEpi cell monolayers
Environment: cell growth media, maintained at 37C, 5% CO_2, >95% relative humidity
Normal force: 250 µN
Contact area ~ 0.44 mm^2
Sliding speed: 1 mm/s
Sliding path length (1/2 cycle) = 3 mm
Total sliding distance: 3.6 m
Number of reciprocating cycles: 600
Duration of sliding experiment: 1 h 21 min
Field of view = 620 µm × 3,000 µm

Objective: 20× (0.62 µm/pixel)
FITC (λ = 488 nm) Laser Power = 0.1; Gain = 14
TRITC (λ = 561 nm) Laser Power = 0.1; Gain = 25
CY5 (λ = 640 nm) Laser Power = 3; Gain = 100

Objective: 4× (3.11 µm/pixel)
FITC (λ = 488 nm) Laser Power = 5; Gain = 25
TRITC (λ = 561 nm) Laser Power = 5; Gain = 50
CY5 (λ = 640 nm) Laser Power = 10; Gain = 100

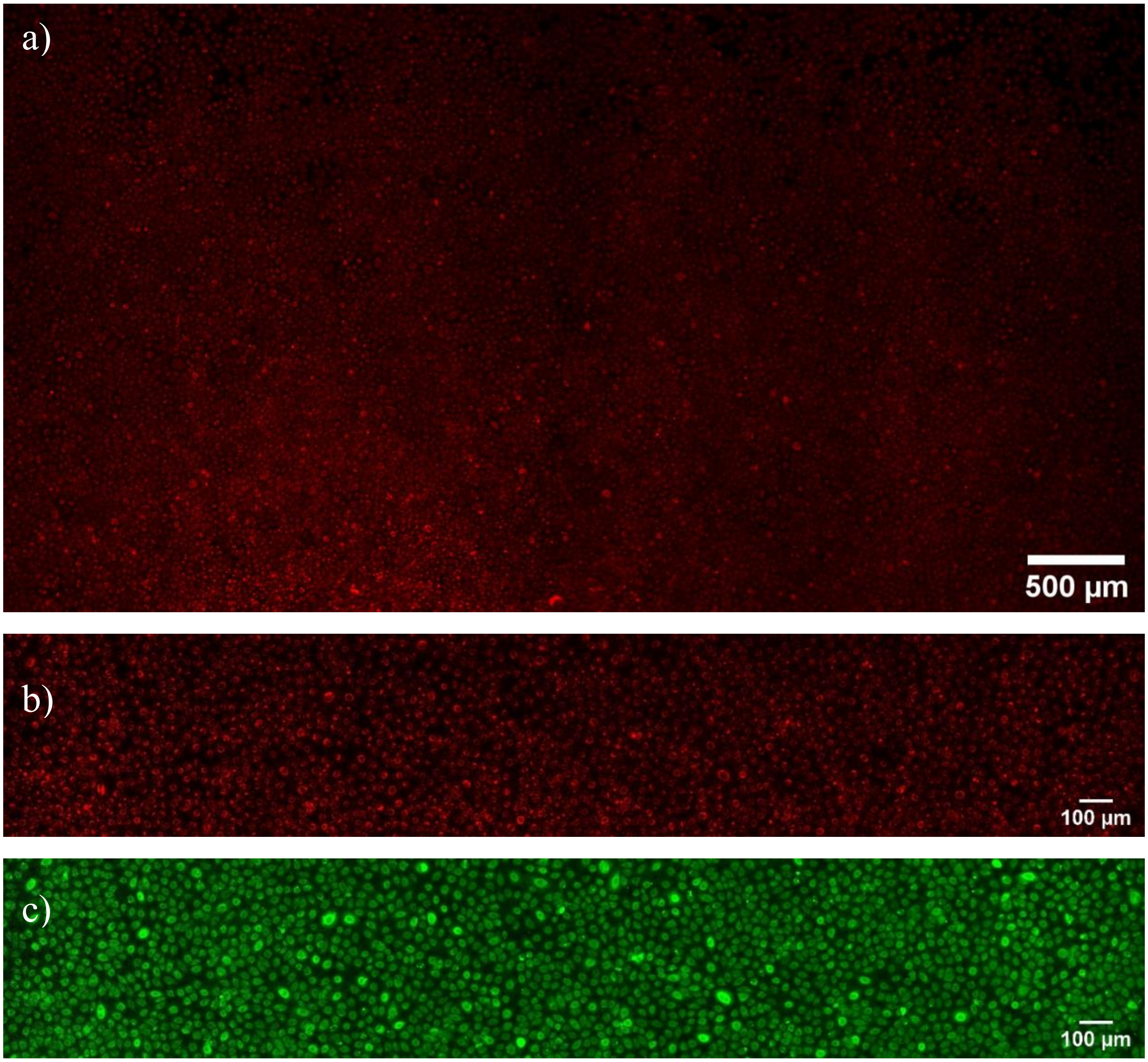

Figure S33. Microscopy images of cells before sliding. a) 4x composite image of cells stained for mucin (red), showing both contacted and non-contacted areas. b) 20x composite image of cells stained for mucin (red) in contact with the probe's sliding path. c) 20x composite image of cells stained with CellTracker™ (green) along the probe's sliding path.

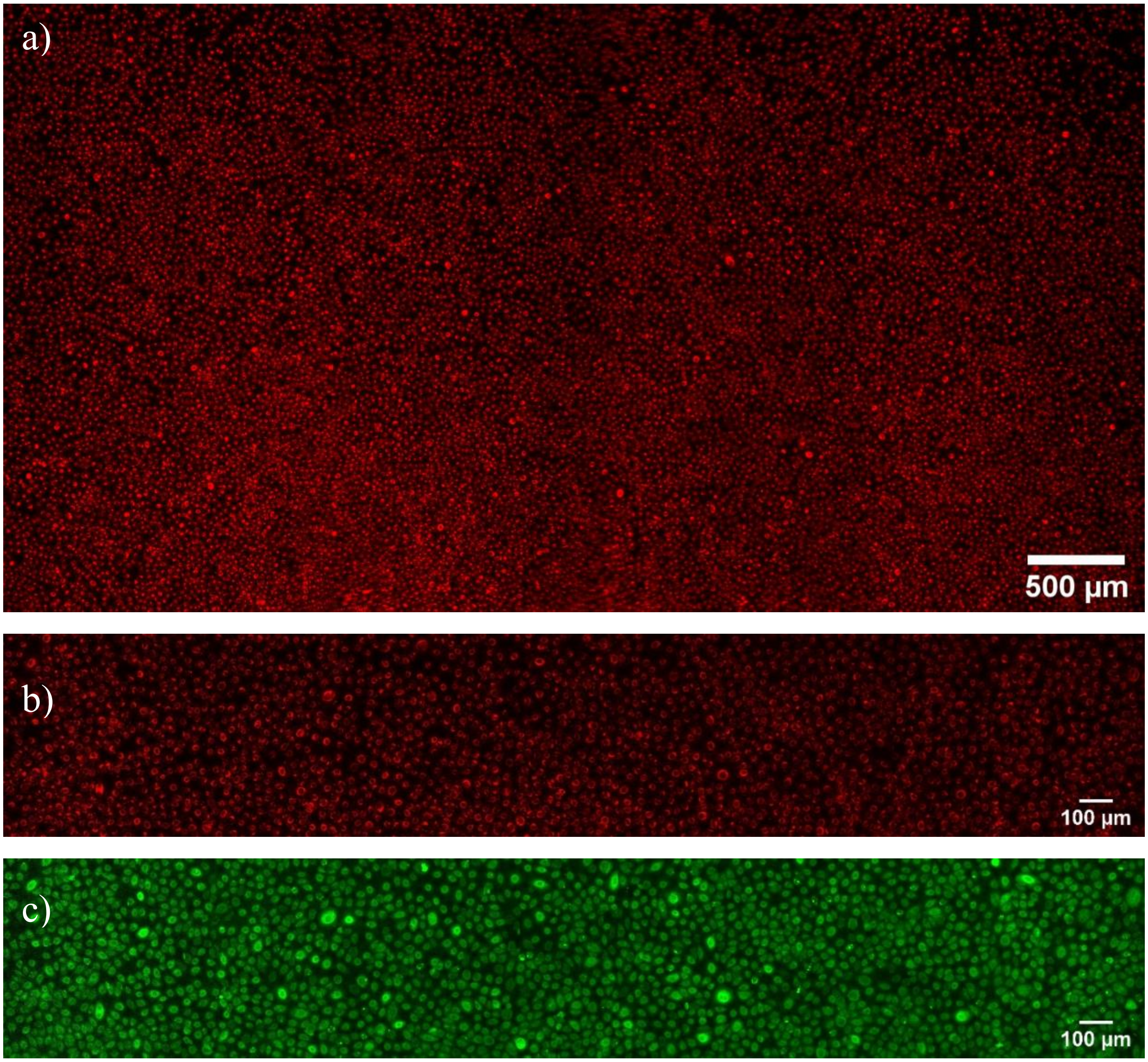

Figure S34. Microscopy images of cells after sliding with a 7.5wt% PAAM gel probe cast against glass. a) 4x composite image of cells stained for mucin (red), showing both contacted and non-contacted areas. b) 20x composite image of cells stained for mucin (red) in contact with the probe's sliding path. c) 20x composite image of cells stained with CellTracker™ (green) along the probe's sliding path.

Experiment 5

Probe: glass-molded polyacrylamide hydrogel
Countersurface: hTCEpi cell monolayers
Environment: cell growth media, maintained at 37C, 5% CO_2, >95% relative humidity
Normal force: 250 μN
Contact area ~ 0.28 mm^2
Sliding speed: 1 mm/s
Sliding path length (1/2 cycle) = 3 mm
Total sliding distance: 3.6 m
Number of reciprocating cycles: 600
Duration of sliding experiment: 1 h 21 min
Field of view = 620 μm × 3,000 μm

Objective: 20× (0.62 μm/pixel)
FITC (λ = 488 nm) Laser Power = 0.1; Gain = 14
TRITC (λ = 561 nm) Laser Power = 0.1; Gain = 50
CY5 (λ = 640 nm) Laser Power = 5; Gain = 100

Objective: 4× (3.11 μm/pixel)
FITC (λ = 488 nm) Laser Power = 5; Gain = 25
TRITC (λ = 561 nm) Laser Power = 5; Gain = 50
CY5 (λ = 640 nm) Laser Power = 10; Gain = 100

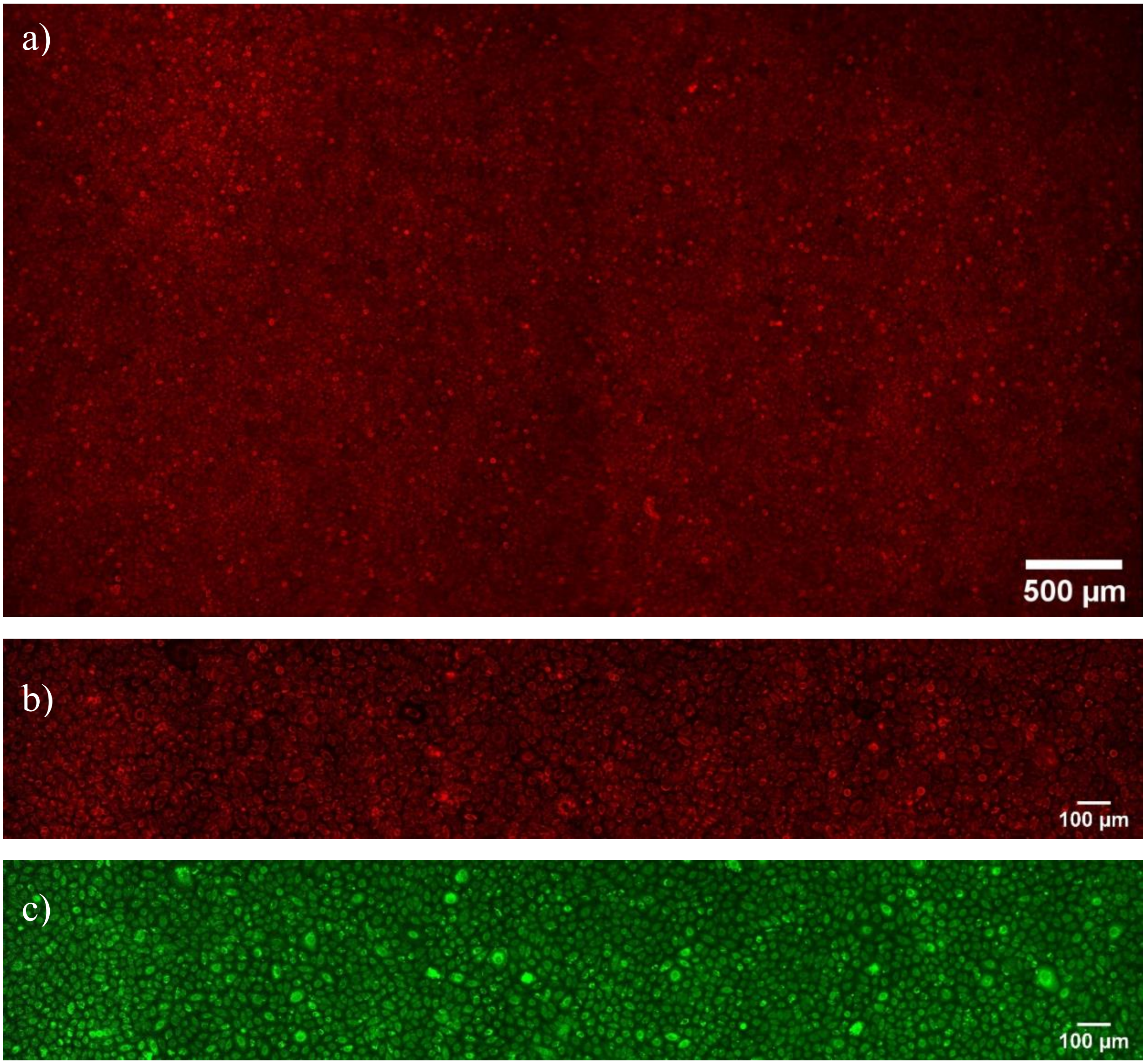

Figure S35. Microscopy images of cells before sliding. a) 4x composite image of cells stained for mucin (red), showing both contacted and non-contacted areas. b) 20x composite image of cells stained for mucin (red) in contact with the probe's sliding path. c) 20x composite image of cells stained with CellTracker™ (green) along the probe's sliding path.

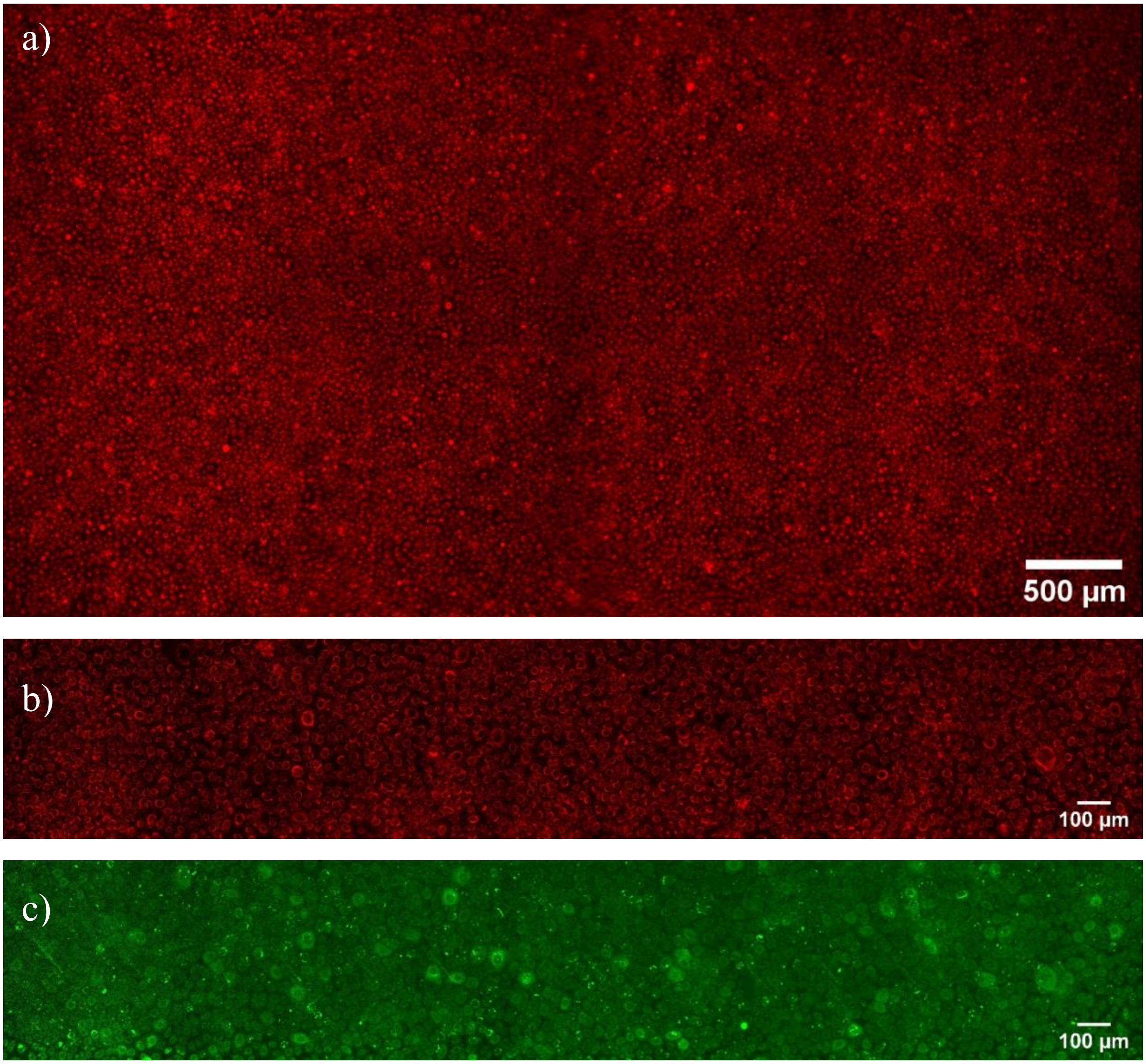

Figure S36. Microscopy images of cells after sliding with a 7.5wt% PAAM gel probe cast against glass. a) 4x composite image of cells stained for mucin (red), showing both contacted and non-contacted areas. b) 20x composite image of cells stained for mucin (red) in contact with the probe's sliding path. c) 20x composite image of cells stained with CellTracker™ (green) along the probe's sliding path.

Experiment 6

Probe: glass-molded polyacrylamide hydrogel
Countersurface: hTCEpi cell monolayers
Environment: cell growth media, maintained at 37C, 5% CO_2, >95% relative humidity
Normal force: 250 μN
Contact area ~ 0.41 mm^2
Sliding speed: 1 mm/s
Sliding path length (1/2 cycle) = 3 mm
Total sliding distance: 3.6 m
Number of reciprocating cycles: 600
Duration of sliding experiment: 1 h 21 min
Field of view = 620 μm × 3,000 μm

Objective: 20× (0.62 μm/pixel)
FITC (λ = 488 nm) Laser Power = 0.1; Gain = 14
TRITC (λ = 561 nm) Laser Power = 0.1; Gain = 50
CY5 (λ = 640 nm) Laser Power = 5; Gain = 100

Objective: 4× (3.11 μm/pixel)
FITC (λ = 488 nm) Laser Power = 5; Gain = 25
TRITC (λ = 561 nm) Laser Power = 5; Gain = 50
CY5 (λ = 640 nm) Laser Power = 10; Gain = 100

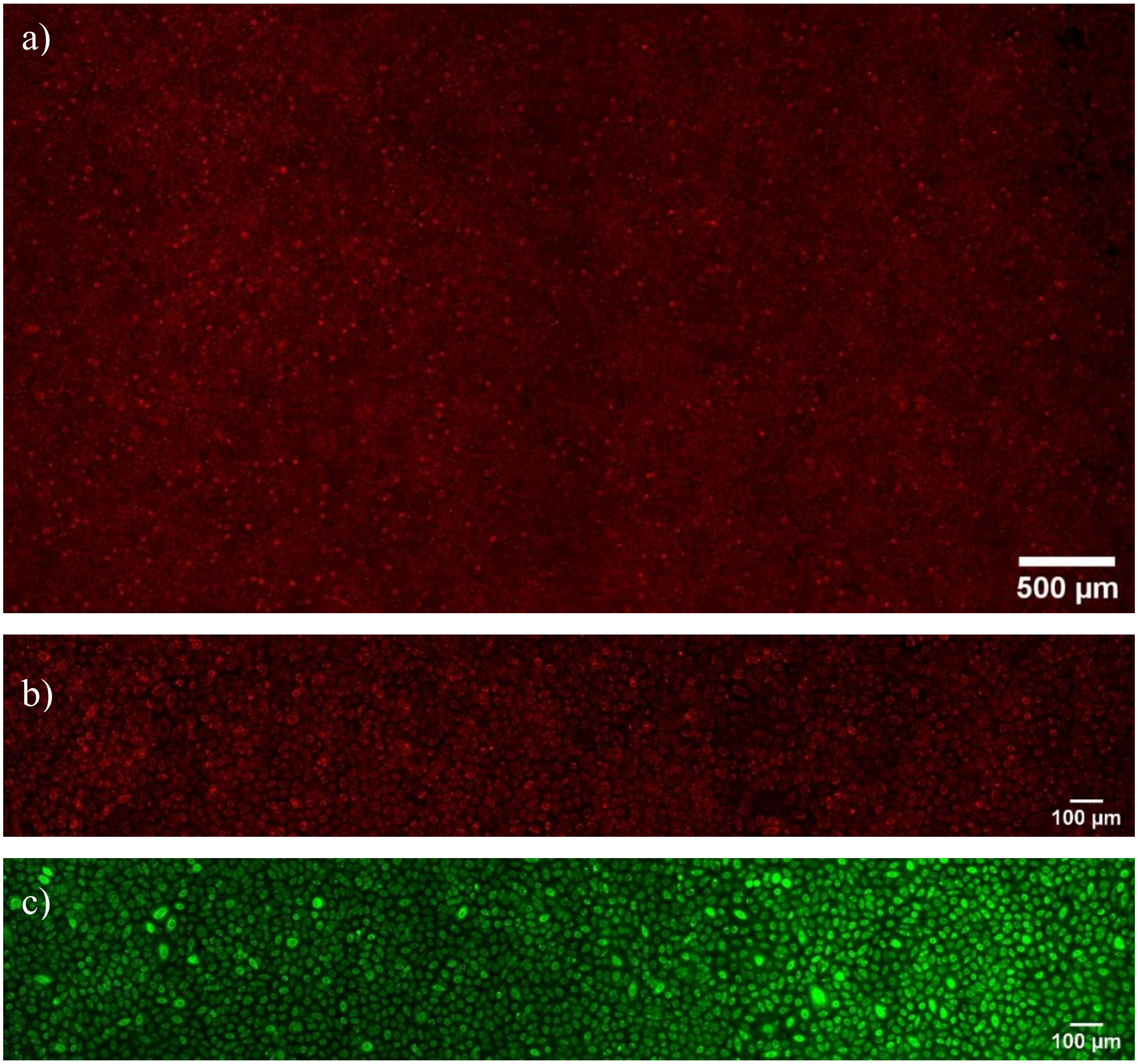

Figure S37. Microscopy images of cells before sliding. a) 4x composite image of cells stained for mucin (red), showing both contacted and non-contacted areas. b) 20x composite image of cells stained for mucin (red) in contact with the probe's sliding path. c) 20x composite image of cells stained with CellTracker™ (green) along the probe's sliding path.

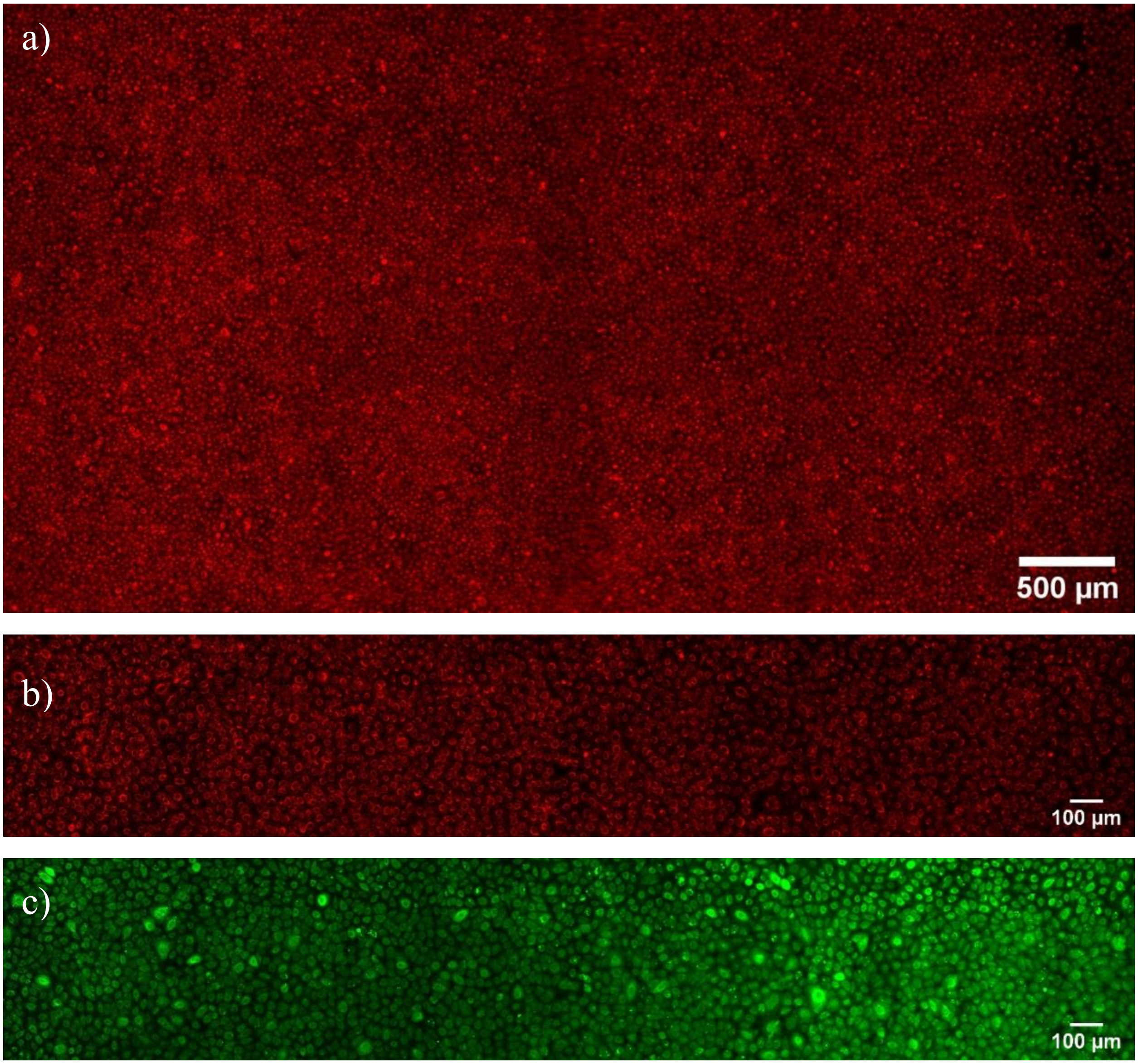

Figure S38. Microscopy images of cells after sliding with a 7.5wt% PAAM gel probe cast against glass. a) 4x composite image of cells stained for mucin (red), showing both contacted and non-contacted areas. b) 20x composite image of cells stained for mucin (red) in contact with the probe's sliding path. c) 20x composite image of cells stained with CellTracker™ (green) along the probe's sliding path.

Section 4: hTCEpi cell monolayers were stained with propidium iodide, at a concentration of 2.39 nM, to assess cell death. Regions of interest, approximately 100,000 µm² in size, were selected to determine cell density. Cell monolayers were examined within and outside of the sliding path. The program cellpose was used to count fluorescent nuclei, indicative of cell death. The difference in the number of dead cells before and after biotribological testing was normalized to the total number of cells within each segment and is provided below as a function of area.

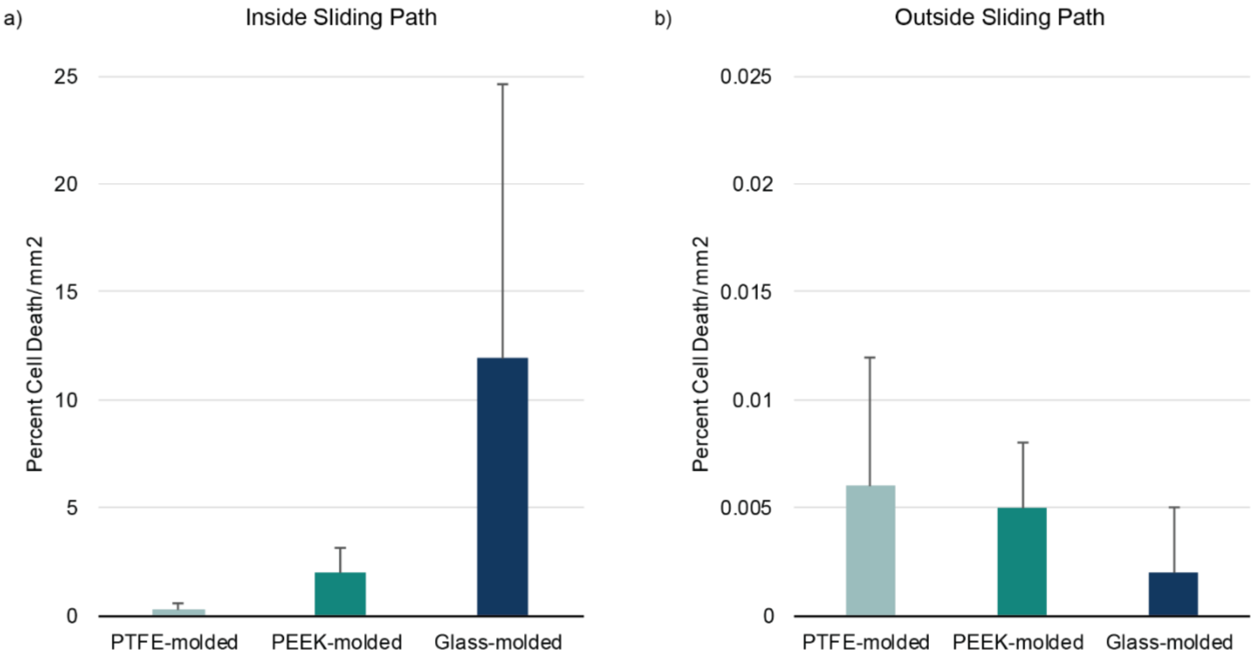

Figure S39. a) Percent of cell death per square millimeter a) within and b) outside of the sliding path. Note that the y-axes are different for a) and b).

Section 5: Elastic modulus measurements of polyacrylamide hydrogels polymerized against glass, PEEK and PTFE using the Optics 11Life Pavone nanoindenter. The nanoindenter was fit with a silica colloidal probe: K_{nano} = 0.025 N/m, tip radius, R_{nano} = 25 µm for PTFE/PEEK-molded gels; K_{nano} = 0.47 N/m, tip radius, R_{nano} = 25 µm for glass-molded gels . Indentation data was fit using Hertzian (red) and Winkler (yellow) contact mechanics models. R^2 values are presented in table form to demonstrate that Hertzian contact mechanics offer a better fit for indentation data of glass-molded gels. Conversely, Winkler contact mechanics exhibit a superior fit for indentation data of PEEK/PTFE-molded gels.

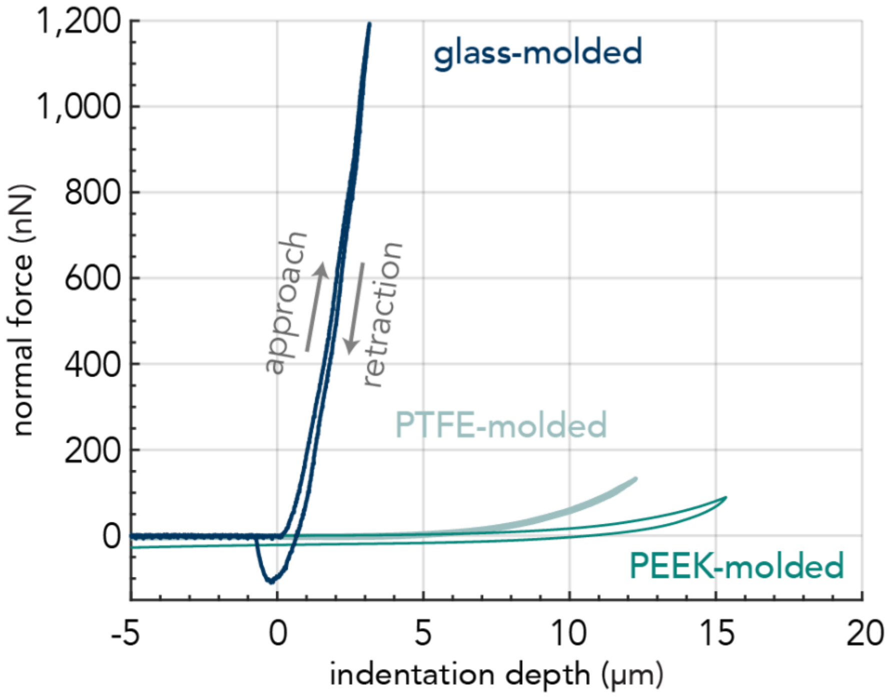

Figure S40. Representative full indentation curves (approach and retraction) shown for glass-(blue), PTFE-(light green), PEEK-molded gels (green).

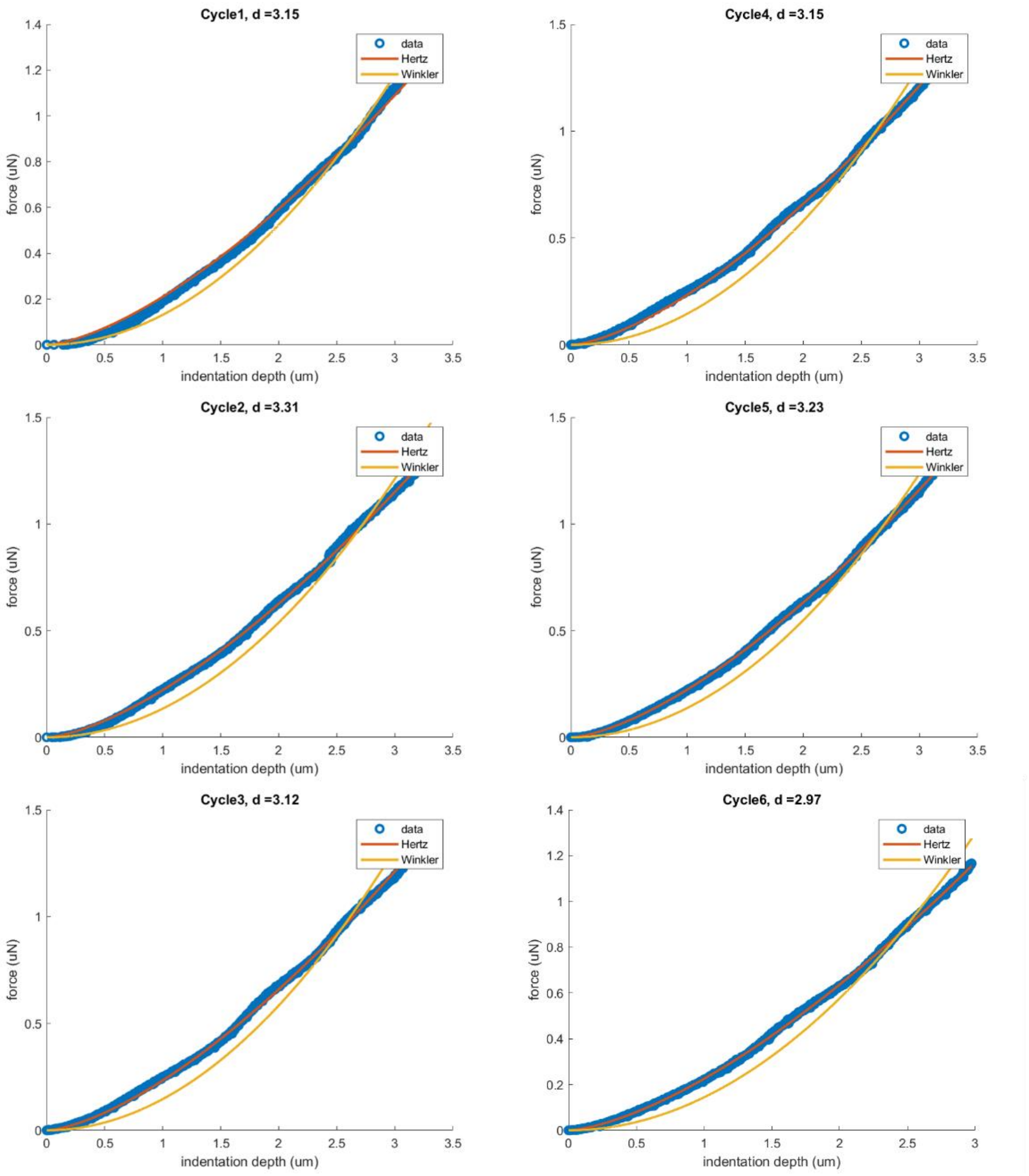

Figure S41. Nanoindentation data (blue) for hydrogel disks polymerized against glass surfaces. Data fit with Hertz (red) and Winkler (yellow) contact mechanics models.

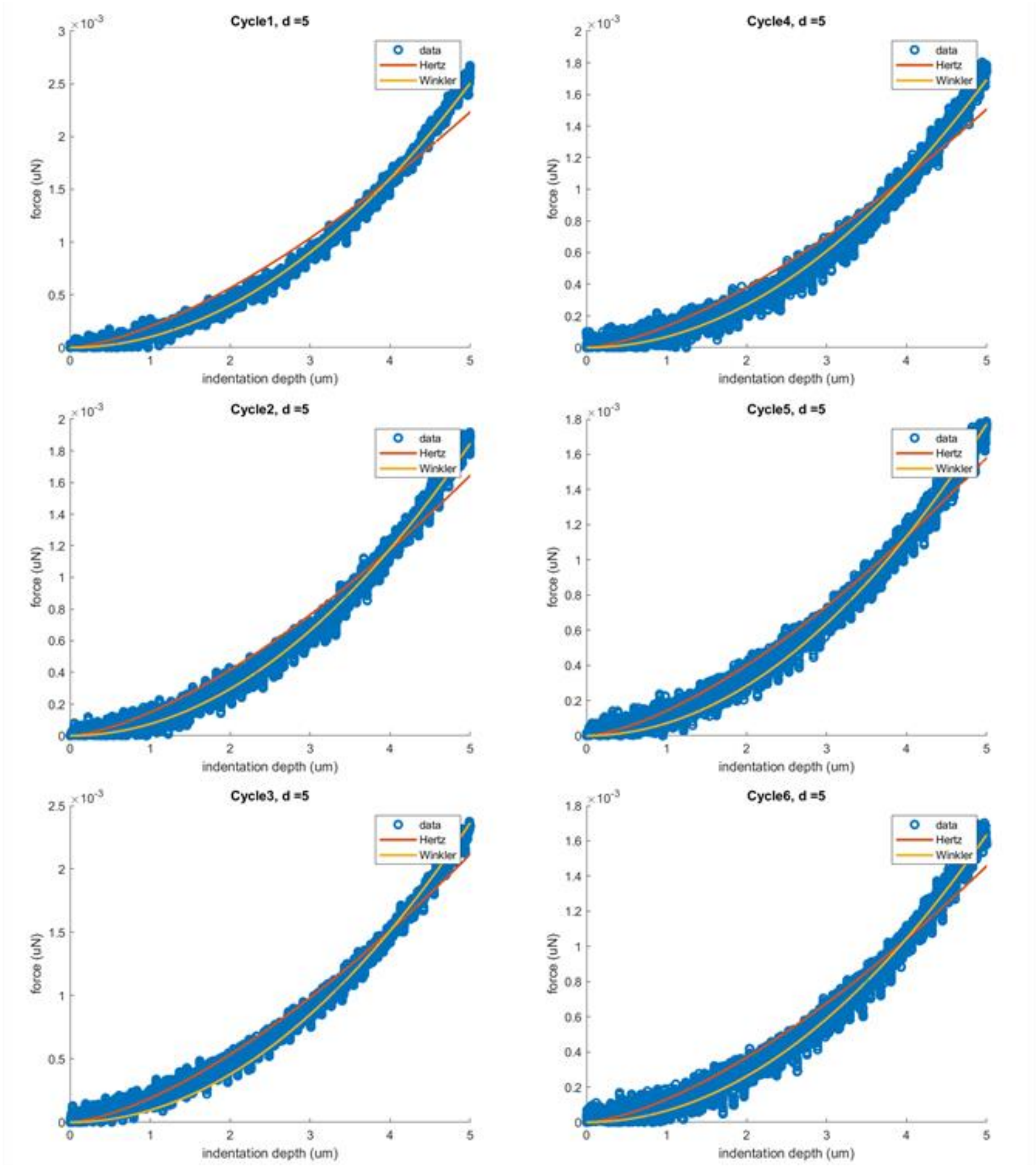

Figure S42. Nanoindentation data (blue) for hydrogels polymerized against PEEK surfaces. Data fit with Hertz (red) and Winkler (yellow) contact mechanics models.

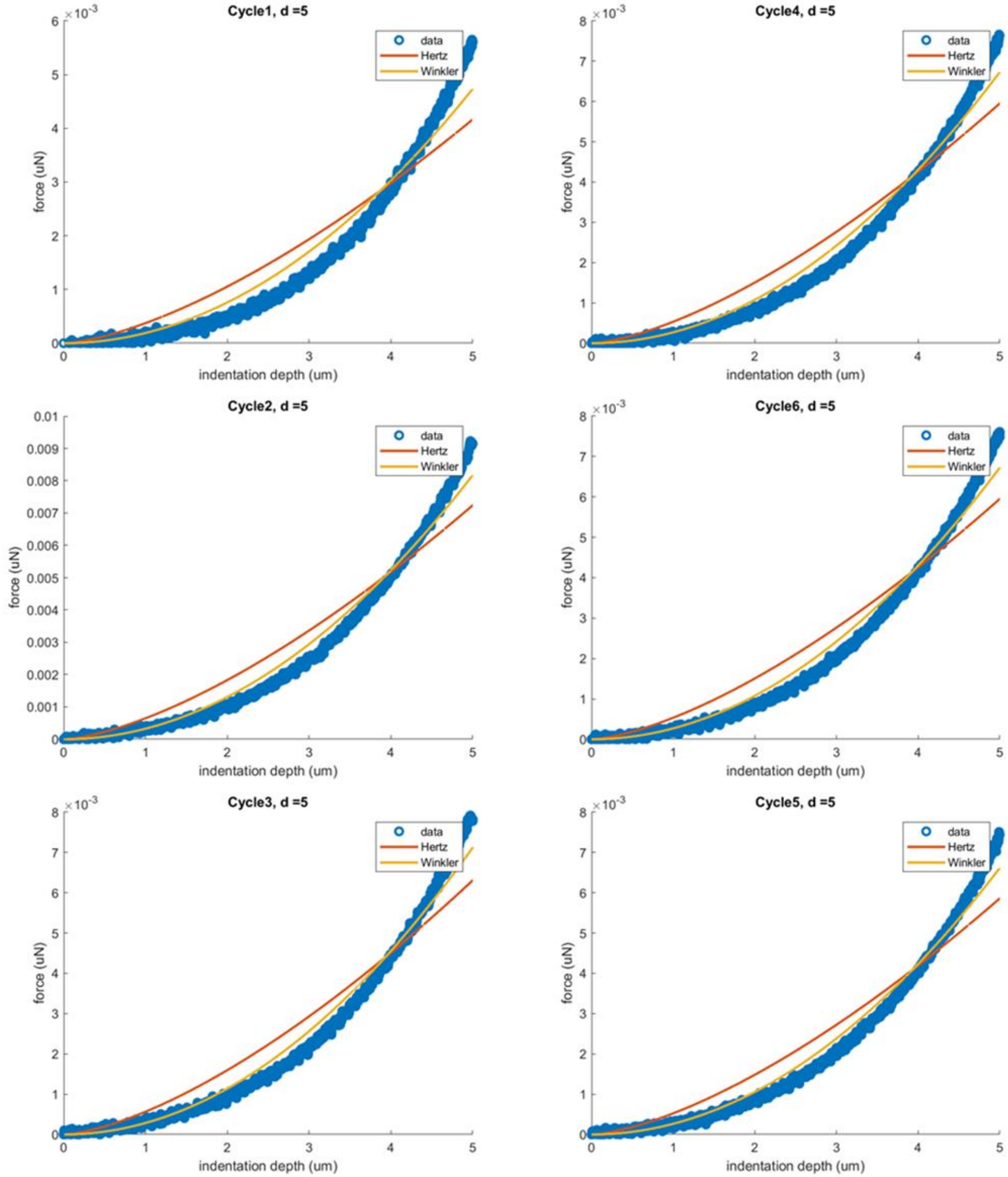

Figure S43. Nanoindentation data (blue) for hydrogels polymerized against PTFE surfaces. Data fit with Hertz (red) and Winkler (yellow) contact mechanics models.

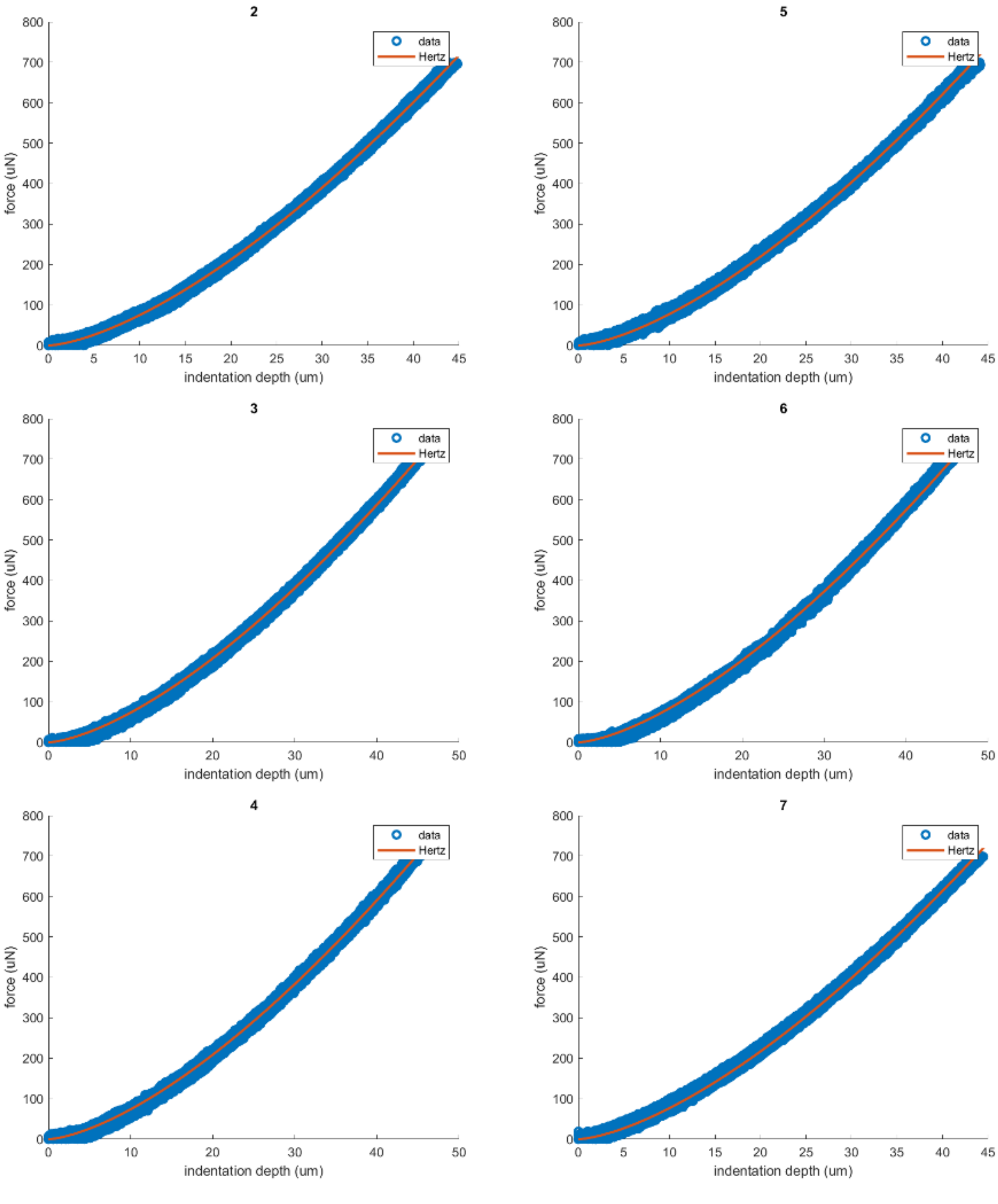

Figure S44. Microindentation data (blue) for hydrogels polymerized against glass surfaces. Data fit with Hertz (red) contact mechanics models.

	Material cast against	Replicate No.	1	2	3	4	5	6
Nanoindentation	Glass	R^2 - Hertz contact mechanics	0.997	0.999	0.999	0.999	0.999	0.999
		R^2 - Winkler contact mechanics	0.980	0.962	0.955	0.954	0.964	0.966
	PEEK	R^2 - Hertz contact mechanics	0.971	0.971	0.986	0.964	0.980	0.973
		R^2 - Winkler contact mechanics	0.996	0.994	0.987	0.991	0.990	0.990
	PTFE	R^2 - Hertz contact mechanics	0.894	0.925	0.930	0.922	0.930	0.924
		R^2 - Winkler contact mechanics	0.964	0.983	0.985	0.981	0.985	0.983
Microindentation	Glass	R^2 - Hertz contact mechanics	0.997	0.998	0.997	0.997	0.997	0.996

Table S1. R^2 values for fits using both Hertz and Winkler contact mechanics models. Winkler contact mechanics models show an improved fit to experimental data for hydrogels with surface gel layers (PEEK- and PTFE-molded surfaces). Conversely, Hertzian contact mechanics models fit better to experimental data in the case of hydrogels cast against glass.

Section 6: Analysis of breakloose friction (static friction) and breakloose shear stress across all experiments.

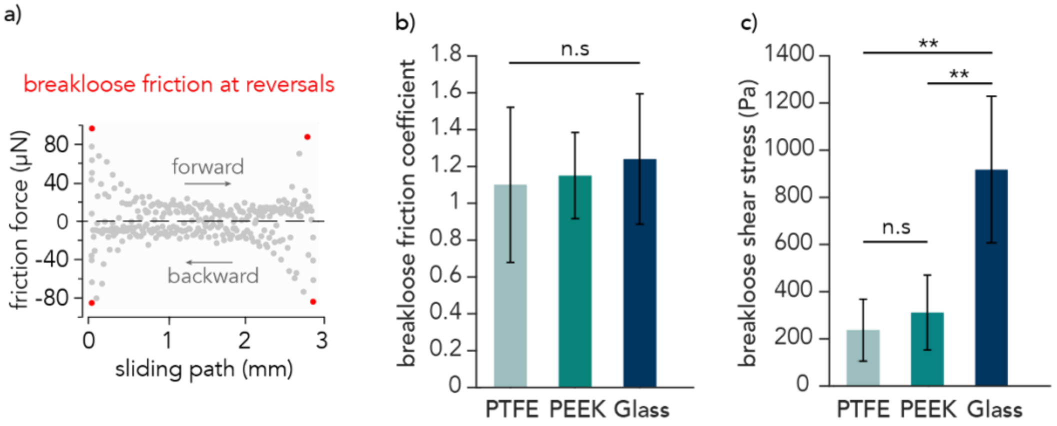

Figure S45. a) Breakloose friction was determined using the maximum friction force recorded at the reversal zones (shown in red). b) Bar plot of the average breakloose friction coefficient calculated from the steady-state regime for glass-molded, PEEK-molded, and PTFE-molded hydrogel probes (N = 6). No statistical significance was found between the gel samples. (c) Bar plot of the average breakloose frictional shear stress for glass-molded, PEEK-molded, and PTFE-molded hydrogel probes (N = 6). There was statistical significance between glass-molded and PEEK-molded (P = 0.0038) and glass-molded and PTFE-molded (P = 0.0002) but not PEEK-molded and PTFE-molded (P = 0.3998)

Section 7: Composite confocal images of glass-,PEEK-, and PTFE-molded probes were taken after completing 600 cycles (3.6 m total). A maximum intensity projection of these images shows fluorescent mucin (red, WGA stain) adhered along the surface of the probe.

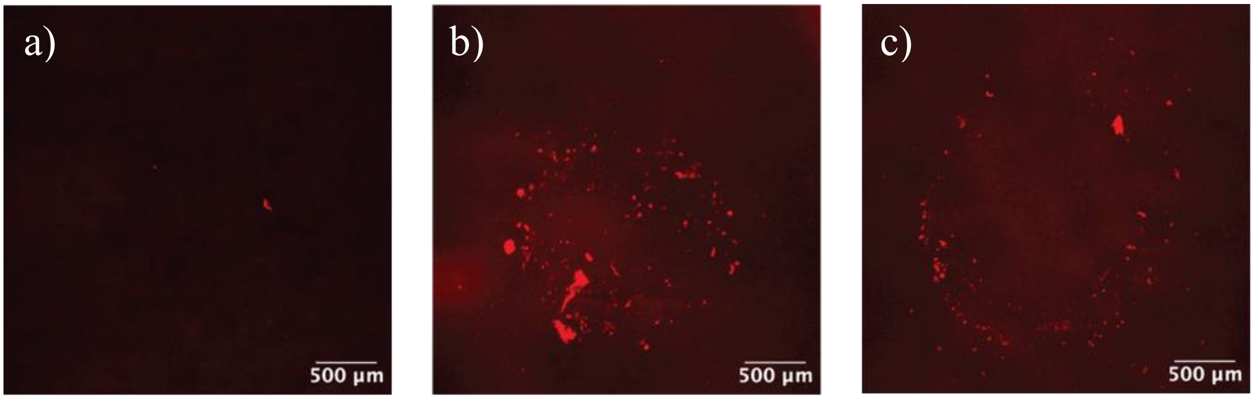

Figure S46. Maximum projections of confocal fluorescence images depicting the apical surfaces of a) glass-, b) PEEK-, and c) PTFE-molded hydrogel probes following 600 cycles of sliding (3.6 m), showing the adhesion of mucin (red, WGA stain) across the probe surfaces.
